# Molecular mechanisms of resistance to *Myzus persicae* conferred by the peach *Rm2* gene: A multi-omics view

**DOI:** 10.3389/fpls.2022.992544

**Published:** 2022-10-05

**Authors:** Pauline Le Boulch, Jean-Luc Poëssel, David Roux, Raphaël Lugan

**Affiliations:** ^1^ UMR Qualisud, Avignon Université, Avignon, France; ^2^ UR GAFL, INRAE PACA, Avignon, France

**Keywords:** Green peach aphid (Myzus persicae), Effector-triggered immunity (ETI), *Prunus persica, Metabolomics*, Pattern-triggered immunity (PTI), Transcriptomics (RNA-Seq), Systemic acquired resistance (SAR), Hypersensitive response (HR)

## Abstract

The transcriptomic and metabolomic responses of peach to *Myzus persicae* infestation were studied in Rubira, an accession carrying the major resistance gene *Rm2* causing antixenosis, and GF305, a susceptible accession. Transcriptome and metabolome showed both a massive reconfiguration in Rubira 48 hours after infestation while GF305 displayed very limited changes. The Rubira immune system was massively stimulated, with simultaneous activation of genes encoding cell surface receptors involved in pattern-triggered immunity and cytoplasmic NLRs (nucleotide-binding domain, leucine-rich repeat containing proteins) involved in effector-triggered immunity. Hypersensitive reaction featured by necrotic lesions surrounding stylet punctures was supported by the induction of cell death stimulating NLRs/helpers couples, as well as the activation of H_2_O_2_-generating metabolic pathways: photorespiratory glyoxylate synthesis and activation of the futile P5C/proline cycle. The triggering of systemic acquired resistance was suggested by the activation of pipecolate pathway and accumulation of this defense hormone together with salicylate. Important reduction in carbon, nitrogen and sulphur metabolic pools and the repression of many genes related to cell division and growth, consistent with reduced apices elongation, suggested a decline in the nutritional value of apices. Finally, the accumulation of caffeic acid conjugates pointed toward their contribution as deterrent and/or toxic compounds in the mechanisms of resistance.

## Introduction

The green peach aphid (GPA), *Myzus persicae*, is a polyphagous sap-sucking pest found throughout the world, attacking many crop species and whose harmful nature is largely due to its ability to transmit plant viruses. The primary host of this aphid is peach (*Prunus persica* L. Batsch), on which it overwinters in the egg stage. Among the peach genetic resources only a few accessions showing strong resistance to GPA have been characterized, all carrying a locus located at the bottom of chromosome 1: “Weeping Flower Peach”, an ornamental genotype carrying *Rm1* (*Resistance to Myzus 1*) gene (Monet and Massonié, 1994; Pascal et al., 2017), Rubira, a red-leaf rootstock carrying *Rm2* (Lambert and Pascal, 2011) and “Fen Shouxing”, a semi-wild selection carrying *Rm3* (Niu et al., 2018). Major aphid resistance loci have also been described and cloned in other species: the root-knot nematode resistance gene *Mi-1.2* (Milligan et al., 1998) confers resistance to *Macrosiphum euphorbiae* in tomato (Rossi et al., 1998; Vos et al., 1998) and *Vat* against *Aphis gossypii* in melon (Dogimont et al., 2014). *Mi-1.2* was the first insect-specific resistance gene to be cloned and the first example of a resistance gene active against distant organisms since it confers resistance to root-knot nematode, *M. euphorbiae*, psyllids (Casteel et al., 2006) and whiteflies (Nombela et al., 2003) in tomato. Furthermore, Pallipparambil et al. (2015) demonstrated an extended spectrum of resistance controlled by this gene, as *Mi-1.2* negatively impacts non-phloem sap-feeding organisms like larvae of *Oirus insidiosus*, which are beneficial zoophytophagous predators that prey on aphids. The *Vat* gene was found to confer dual resistance to *Aphis gossypii* and to viruses it transmits (Dogimont et al., 2014). This resistance involved a localised hypersensitive response (HR) (Villada et al., 2009), negatively influenced aphid nutrition and has been shown effective against most *Aphis gossypii* clones (Boissot et al., 2016a; Boissot et al., 2016b).

*Mi-1.2* and *Vat* have been found to encode effector-triggered Immunity (ETI) receptors, *i.e.* resistance proteins (R) containing remarkable Nucleotide-Binding and Leucine-Rich Repeat domains (NLR or NBS-LRR) (see Ngou et al., 2022a and Ngou et al., 2022b for review on NLRs). These immunity proteins are divided into three classes according to their N-terminal domain: Toll/interleukin-1 receptor (TNLs), coiled coil domain (CNLs) or RPW8-like coiled coil domain (RNLs) (Jones et al., 2016). Both *Mi-1.2* and *Vat* were characterised as CNLs (Dogimont et al., 2014) and a strong TNL candidate for peach *Rm3* has been recently identified (Pan et al., 2022). ETI is based on intracellular NLR sensors acting alone or in pairs (Feehan et al., 2020) and is one of the two major layers of the plant immune system. The other layer is the pattern-triggered Immunity (PTI), made up of pattern recognition receptors (PRRs), *i.e.* cell surface sensors activated by pathogenesis-associated molecular patterns (PAMPs), herbivore-associated molecular patterns (HAMPS) or damage-associated molecular patterns (DAMPS). PRRs include receptor-like kinases (RLKs) and receptor-like proteins (RLPs), which comprise a variable extracellular domain allowing the recognition of various ligands, a transmembrane domain and a cytoplasmic kinase in the case of RLKs (Tang et al., 2017) which transmits a signal to intracellular proteins once activated. The selection pressure exerted by PTI on pathogen populations has led to the emergence of populations carrying effector proteins that neutralize resistance mechanisms and restore virulence through Effector Triggered Susceptibility (ETS, Ngou et al., 2022a). This, in turn, resulted in the evolution of plant populations that carry cytosolic receptors capable of specifically recognize pathogen effectors and initiating the strong defenses that constitute ETI (Jones and Dangl, 2006).

Aphids are piercing-sucking insects that feed exclusively on phloem sap, which they collect with their specialized mouthpiece, the stylet. Their feeding behavior comprises several phases generating signals capable of triggering plant defense: the stylet and the secreted gel-like saliva can produce PAMPs and DAMPs during intercellular insertion and mesophyll cells probing phases, while the watery saliva secreted when sucking phloem sap from sieve elements contains protein effectors that suppress plant defense, (Will et al., 2007; Bos et al., 2010), contributing to the stealthy nature of aphids, but that may potentially trigger ETI. Indeed, defense against aphids proved to involve both PTI and ETI. The role of PTI in defense against aphid infestation was demonstrated by application of eliciting extracts of GPA (Prince et al., 2014) and by the study of the Arabidopsis *bak1* mutant, deficient in the *BAK1* RLK coreceptor (Prince et al., 2014; Chaudhary et al., 2014; Tungadi et al., 2021).

Omic studies have uncovered other molecular constituents of plant-sap-sucking insects including aphids interplay (reviewed by Zogli et al., 2020) and many of them concern the model *Arabidopsis thaliana*-GPA interaction (Louis and Shah, 2013). Kerchev et al. (2013), for example, showed the importance of redox control, SA and abscisic acid (ABA) signalling pathways in Arabidopsis after infestation by GPA. Analysis of transcriptional responses to *Macrosiphum euphorbiae* in tomato also highlighted the involvement of antioxidant mechanisms and hormonal regulations: SA, jasmonic acid (JA), ethylene and brassinosteroids (BRs) and the activation of callose synthesis genes, dedicated to blocking symplastic connections (Kuśnierczyk et al., 2008; Coppola et al., 2013). In peach, a transcriptomic study of “Fen Shouxing” after GPA infestation revealed the activation of thousands of genes involved in signalling cascades or resistance mechanisms, such as redox modifications, calcium fluxes, mitogen-activated protein kinases (MAPKs), phytohormones, transcription factors, pathogenesis-related proteins (PRs) and enzymes of secondary metabolic pathways (Niu et al., 2018). Metabolomic studies on plant-aphid interactions are less common but crucial to uncovering metabolites involved in stress signalling and defense (Zogli et al., 2020) as well as reconfigurations of carbon and nitrogen fluxes controlling the growth and nutritional value of infested plants. Gao et al. (2021) reported alterations in central and secondary metabolism of rose in response to *Macrosiphum rosivorum* and Kuśnierczyk et al. (2008) confirmed the antibiotic role of secondary metabolites such as glucosinolates and the alkaloid camalexin in Arabidopsis.

Molecular responses of Arabidopsis to GPA have been extensively studied, but ecotypes of this secondary host exhibit only partial antibiosis and antixenosis (Cabrera y Poch et al., 1998) and major R genes capable of triggering ETI have never been described in this species. In this respect, peach, as primary host of GPA with lines owning *Rm* genes conferring total antixenosis, is a relevant species to decipher all layers of the plant immune mechanisms involved in strong resistance to these phloem-feeders. In the present study, we establish a detailed picture of signal transduction and metabolic mechanisms triggered by activation of *Rm2* upon GPA infestation. We analyzed the responses of Rubira, carrying *Rm2*, and compared it to the susceptible accession GF305, after 48 hours of infestation, a duration required and sufficient to trigger induced resistance in Rubira (Sauge et al., 2011). We evaluated the global metabolic and transcriptomic reprogramming and looked for differentially expressed genes involved in PTI and ETI, hypersensitive response (HR) and systemic acquired resistance (SAR). We also analyzed metabolic reconfigurations underlying the production of hormonal signals and defense compounds and examined pathways reflecting a reorientation of growth fluxes and activation of oxidative burst.

## Materials and methods

### Plant material

Two highly homozygous seedling rootstocks selected by INRAE and contrasting for their resistance to GPA were used: the susceptible rootstock GF305 and the red-leaf rootstock Rubira, carrying the dominant resistance gene *Rm2* (Lambert & Pascal, 2011). Seeds were produced by natural inbreeding in isolated orchards of a commercial nursery (Pépinières Lafond, Valréas, France). Disinfected seeds were placed in petri dishes containing humidified perlite and were stratified during 3 months in dark cold room at 4°C. After radicle emergence, seeds were sown, grown in a greenhouse and kept free of pests and diseases without spraying or biological control. Axillary shoots were removed to keep only the main stem. 8 weeks after sowing, 10 plants of each genotype were acclimated during one week in the air-conditioned room where experiments took place, at 22°C with a 16 h day/8 h night photoperiod. The pots were placed in large trays filled with water to prevent the movement of aphids from one plant to another during the experiment.

### Aphids

The *Myzus persicae* clonal line used, Mp06, was obtained in 2013 from a single egg collected in a peach orchard (Avignon, France). Since then, parthenogenetic apterous females were continuously reared on GF305 seedlings in an air-conditioned room at 22°C with 16h day/8h night photoperiod.

### Infestation and plant sampling

One week before infestation, parthenogenetic females were installed on GF305 seedlings for laying and removed after 48 hours to obtain a nymph population of the same age. The day of infestation, the synchronized adult females obtained were placed for 4 h at 22°C to generate a fasting period. Ten plants of GF305 and Rubira were infested by placing carefully with a brush 10 aphids on their apex. After 48h, aphids and nymphs were pulled out with a brush, control plants (10 for each genotype) were equally stimulated with the brush, and the apices of control and infested plants were cut below the second elongated internode (approximately 100 mg of fresh material per plant), immediately frozen in liquid nitrogen and then stored at -80 C until analysis. Each apex was analysed individually and a set of 4 replicates were dedicated to transcriptomic analysis and another 5 to metabolomics.

A second experiment was conducted in the same conditions for a kinetic study of phytohormones with harvest of apex 12, 24 and 48 hours post-infestation (hpi) and for measure of plant elongation 7 days post-infestation (dpi).

### RNA extraction and sequencing

Each sample was manually ground in liquid nitrogen with disposable pestles and total RNA was extracted with the RNeasy Plant Mini Kit (QUIAGEN, France) according to the manufacturer’s instructions, which comprises a guanidine isothiocyanate lysis and purification with silica membrane. RNA concentration and purity were checked with a Nanodrop (Thermo Fisher Scientific, Wilmington, USA), a QuBit 3.0 Fluorometer (Thermo Fisher Scientific, Wilmington, USA) and RNA integrity with a Bioanalyzer 2100 (Agilent Technologies, Santa Clara, USA). Next generation sequencing of RNA (RNAseq) was realized by the GeT-PlaGe platform (INRAE, Toulouse, France). Sixteen RNAseq libraries were prepared with the mRNA TruSeq Stranded kit (Illumina, San Diego, USA), according to Illumina’s protocols. Briefly, mRNAs were isolated from total RNA using poly-T beads, fragmented and converted to cDNA. Specifics adapters and multiplexing indexes were ligated before PCR amplification. Libraries quality was checked using a Fragment Analyser (AATI, Ankeny, USA) and they were quantified by qPCR using Quant Studio 6 Real-Time PCR system (Thermo Fisher Scientific, Wilmington, USA). Finally, libraries were pooled on a single flowcell line (Illumina HiSeq3000 sequencer) for paired-end sequencing (2 x 150 bp).

### RNAseq analysis

Sequencer output raw data files (binary base call format) were converted to Fastq format using the CASAVA (Consensus Assessment of Sequence And VAriation) Illumina software. The global quality of sequences was assessed using FastQ Illumina filter and FastQC software (Babraham Institute, Cambridge, UK). Sequencing adapters were removed using Cutadapt version 1.14 (Martin, 2011). Then, the reads were splice-aligned on the peach reference genome version 2.0.a1 (Verde et al., 2017) using STAR version 2.5.1b software (Dobin et al., 2013). Finally, transcript expression was quantified using RSEM version 1.3.0 (Li and Dewey, 2011).

The raw sequencing data were filtered by excluding transcripts with zero or less than 8 counts (corresponding to at least one count for half of the samples), thus reducing the number of transcripts detected to 20606, out of the 26873 protein-coding genes predicted in version 2.0.a1 of the peach reference genome. To evaluate the statistically significant changes in gene expression, an “Independent hypothesis weighting” test was applied according to the DESeq2 R package protocol, with a max fold change of 2, a p.value threshold set to 0.05 and an adjusted false discovery rate (FDR). To overcome the poor annotation of the peach transcriptome, we performed a blastp of the full peach proteome version 2.0.a1 against *Arabidopsis thaliana* Araport11 protein sequences. The set of best Arabidopsis homologs obtained for each peach protein was used to conduct overrepresentation tests and enrichment of expression data *via* panther.org (Thomas et al., 2003) and gProfiler (Raudvere et al., 2019) through the Gene Ontology (GO) and KEGG (Kyoto Encyclopaedia of Genes and Genomes) databases. A *P. persica* protein was considered as the “true” ortholog of an Arabidopsis protein when it was also the best match of a reverse blastp (from *Arabidopsis* to *P. persica* proteomes); a total of 13097 Arabidopsis orthologs were found. Detailed gene function was also retrieved from The Universal Protein Resource (UniProt) and The Arabidopsis Information Resource (TAIR) online databases (Berardini et al., 2015; The UniProt Consortium, 2021). Enrichment analysis was conducted using fold enrichment cut-off of 1.5 and p-value cut-off of 0.05. Fold enrichment is the ratio between the frequency of term genes in a given list (here the lists of induced or repressed genes) and the frequency of term genes expected in this list, based on the frequency in the reference list (complete list of annotated genes). The p-value is the probability that the number of term genes observed occurred by chance (randomly), as determined by the reference list. The full dataset of transcripts can be found in **Supplementary Table S1** and the raw (BioProject ID PRJNA877419) is available in the NCBI Sequence Read Archive.

### Metabolites extraction

Solvents were purchased from Honeywell (Charlotte, USA) and Thermo Fisher Scientific (Wilmington, USA) and ultra-pure water was obtained from a Milli-Q system (Merck, Darmstadt, Germany). Frozen fresh samples were ground in 2 mL microtubes with ball mills for 1 min at 30 Hz (Mixer mill MM200, Retsch, Eragny, France), then 10 mg of fresh powder was extracted for 15 min at 70°C under stirring (940 rpm) with 1.5 mL of a methanol/water (80:20, v/v) solution containing 50 µM of ribitol as internal standard. Volume was adjusted to keep the same ratio mass to volume in every sample. Samples were centrifuged 5 min at 26 400 g and the supernatants containing polar metabolites were filtered before analysis (0.22 µm filters Millex-Lg, PTFE hydrophile, 4 mm, Sigma Aldrich, Merck, Darmstadt, Germany).

### Untargeted GC-EI-TOFMS profiling

The method was adapted from Roessner et al. (2000). Samples were derivatized online before injection with a MultiPurpose Sampler (Gerstel MPS, CTC Analytics AG, Mülheim an der Ruhr, Switzerland): dry extracts were incubated in 50 µL of a pyridine solution containing 20 mg/mL of methoxyamine hydrochloride under constant shaking at 900 rpm and 80°C for 90 min. Then, 80 µL of BSTFA containing a mixture of 9 n-alkanes were added before heating for 30 min at 80°C under constant shaking at 900 rpm. Data acquisition was performed with a gas chromatograph system (7890B GC, Agilent Technologies, Santa Clara, CA, USA) equipped with a capillary column (ZB-SemiVolatiles, 34.59 m, internal diameter 250 µm, film thickness 250 µm, Phenomenex, Torrance, USA) hyphenated to a time-of-flight (TOF) mass spectrometer (Pegasus BT, Leco, Saint Joseph, Benton Harbor, MI, USA). One microliter of sample was injected in split mode (1:50) at 230°C. Helium was used as carrier gas at 0.6 mL/min. The initial oven temperature was kept at 70°C for 1 min and then increased to 320°C (9°C/min) and maintained for 10 min. The m/z scan range was 70–600 with a cycle time of 20 scans/s. Source temperature and transfer line were set at 250°C. The MultiPurpose Sampler was controlled by Maestro Version 1.4.40.1. Gerstel and gas chromatography system with mass spectrometer were controlled by ChromaTOF Version 5.20.38.0.54864 (LECO, Saint Joseph, MI, USA). GC–EI–TOFMS data were deconvoluted with the LECO NTD software (LECO, St.Joseph, MI, USA), then the peak list was curated manually to remove incorrectly deconvoluted peaks and contaminants. Peak annotation was based on spectral and retention index (RI) similarity using mass spectral libraries (Golm database, NIST 2014, Leco-fiehn rtx5). Identification level of each metabolites was determined according to criteria inspired by Schymanski et al. (2014): level 1, confirmed structure by comparison with authentic standard, reverse match > 850, difference between retention index (RI) < 1%; level 2, probable structure, reverse match > 800 and difference between RI < 1%; level 3, tentative candidate, 600 < reverse match < 1000 and difference between RI > 1%. A specific extracted ion chromatogram (XIC) was chosen for each molecule for integration; then peak areas were normalized against the internal standard so the final dataset consisted of semi-quantitative information. The list of metabolites detected and their analytical features can be found in **Supplementary Table S2**; raw dataset (accession number MSV000084377) can be downloaded from the publicly available MassIVE repository at the UCSD Center for Computational Mass Spectrometry website.

### Untargeted UPLC-ESI-QTOF-MS/MS profiling

Analyses were performed with an Acquity I-Class UPLC system (Waters, Mildorf, MA) hyphenated to a Synapt G2-Si quadrupole time-of-flight (QTOF) mass spectrometer (Waters, Mildorf, MA) equipped with an electrospray ionization source (ESI). Chromatographic separation was achieved using a Kinetex 1.7 µm F5 Core-shell LC columns (150 x 2.1 mm, Phenomenex, California, USA). The mobile phase consisted of water (A) and acetronitrile (B), both containing 0.1% formic acid. One microliter of sample was injected before running the solvent gradient: 2% B for 1 min, then up to 100% B in 18 min followed by 2 min at 100% B and then back to initial conditions in 1 min (total run time 23 min). The column was maintained at 35°C with a flow rate of 0.3 mL/min. The source temperature was set to 120°C and the desolvation temperature to 600°C. The capillary voltage was set to 0.8 kV and the cone voltage to 40 V. Nitrogen was used as the drying and nebulizing gas, with 50 L/h gas flow and 800 L/h desolvation gas flow. Analysis was performed twice, in negative and positive ionization modes, with a resolution of 40 000. Data independent acquisitions were performed simultaneously in MS and MS/MS modes (MS^e^, continuum), with a collision energy ramp from 20 V to 70 V. Mass spectra were recorded at 0.2 second per scan from 50 to 1200 m/z. Chromatograms from negative mode acquisition were scanned to extract the major compounds. Metabolites were identified at the level 3 (Schymanski et al., 2014) by a manual examination of MS and MS/MS spectra in positive and negative modes and spectral comparison with in-house database and literature (**Supplementary Table S3**).

### Targeted UPLC-DAD-ESI-TQMS profiling

Analyses were performed with an Acquity I-Class UPLC system (Waters, Mildorf, MA) equipped with a diode array detector (DAD) and hyphenated to a Xevo TQ-XS (Waters, Mildorf, MA) equipped with an ESI source. Pure standards (Merck KGaA, Darmstadt, Germany) were injected and MRM (multiple reactions monitoring) methods were optimized by testing ESI polarity, cone voltage and collision energy. Data acquisition and analysis were performed in using MassLynx software (Waters, Mildorf, USA). Identification level of each metabolites was determined according to Schymanski et al. (2014) as follows: level 1, confirmed structure by comparison with authentic standard; level 2, probable structure by comparison with data bases and/or literature; level 3, MS^2^ spectrum interpretation and light absorbance spectrum matched a tentative candidate. List of metabolites with detailed analytical parameters are provided in **Supplementary Table S4**.

#### Phenolic compounds

Chromatographic separation was achieved using a Kinetex 1.7 µm F5 Core-shell LC columns (150 x 2.1 mm, Phenomenex, California, USA). The mobile phase consisted of water (A) and acetronitrile (B), both containing 0.1% formic acid. One microliter of sample was injected before running the solvent gradient: 2% B for 1 min, then up to 55% B in 18 min, 1 min to reach 100% B followed 2 min at 100% B and then back to initial conditions in 3 min (total run time 25 min). The column was maintained at 35°C with a flow rate of 0.25 mL/min. The source temperature was set to 120°C and the desolvation temperature was set to 600°C. The capillary voltage was set to 1.2 kV. Nitrogen was used as the drying and nebulizing gas, with 50 L/h gas flow and 800 L/h desolvation gas flow.

#### Amino acids and polyamines

Polyamines and amino acids were analyzed after derivatization with 6-Aminoquinolyl-*N*-hydroxysuccinimidyl carbamate (AccQ-Tag Ultra Derivitization Kit, Waters, Mildorf, USA) according to the manufacturer’s instructions. Chromatographic separation was achieved using an Acquity UPLC BEH C18 1.7 µm column (2.1 x 50 mm, Waters, Mildorf, USA) with a pre-column. One microliter of sample was injected before running the solvent gradient: 0.01% B for 0.54 min, then up to 9.1% B in 6.5 min, 2 min at 21.2% B followed by 0.4 min at 59.6% B and then back to initial conditions in 0.6 min (total run time 10.1 min). The column was maintained at 55°C with a flow rate of 0.7 mL/min. The source temperature was set to 150°C and the desolvation temperature was set to 650°C. The capillary voltage was set to 3.0 kV. Nitrogen was used as the drying and nebulizing gas, with 600 L/h gas flow and 1200 L/h desolvation gas flow.

#### Kinetic analysis of salicylic acid and salicylic acid glucoside

One milliliter of polar extract was evaporated under vacuum overnight. The dry residue was concentrated in 100 µL of a methanol/water (80:20, v/v) solution. Chromatographic separation was achieved using an Acquity 1.7 µm C18 CSH LC column (100 x 2.1 mm, Waters, Mildorf, USA). The mobile phase consisted of water (A) and methanol (B), both containing 0.01% formic acid. Five microliters of sample were injected before running the solvent gradient: 25% B for 0,5 min, then up to 100% B in 6 min followed 2 min at 100% B and then back to initial conditions in 1,5 min (total run time 9,5 min). The column was maintained at 45°C with a flow rate of 0.4 mL/min. The source temperature was set to 120°C and the desolvation temperature was set to 550°C. The capillary voltage was set to 2.8 kV. Nitrogen was used as the drying and nebulizing gas, with 150 L/h gas flow and 1000 L/h desolvation gas flow.

### Data analysis

Boxplots were generated with the R packages “PMCMRplus” and “ggoplot” and statistically significant differences were found with a Kruskal-Wallis test and Conover *post-hoc* test with Bonferroni adjustment. Principal component analysis (PCA) and Heatmap were generated with the R package “FactoMineR” and “Pheatmap” respectively, after performing log transformation and Pareto scaling. Metabolites were mapped to metabolic pathways using the Plant Metabolic Network database (Hawkins et al., 2021).

## Results

### Response to infestation: Aphid behavior and plant symptoms

**Figure 1:**
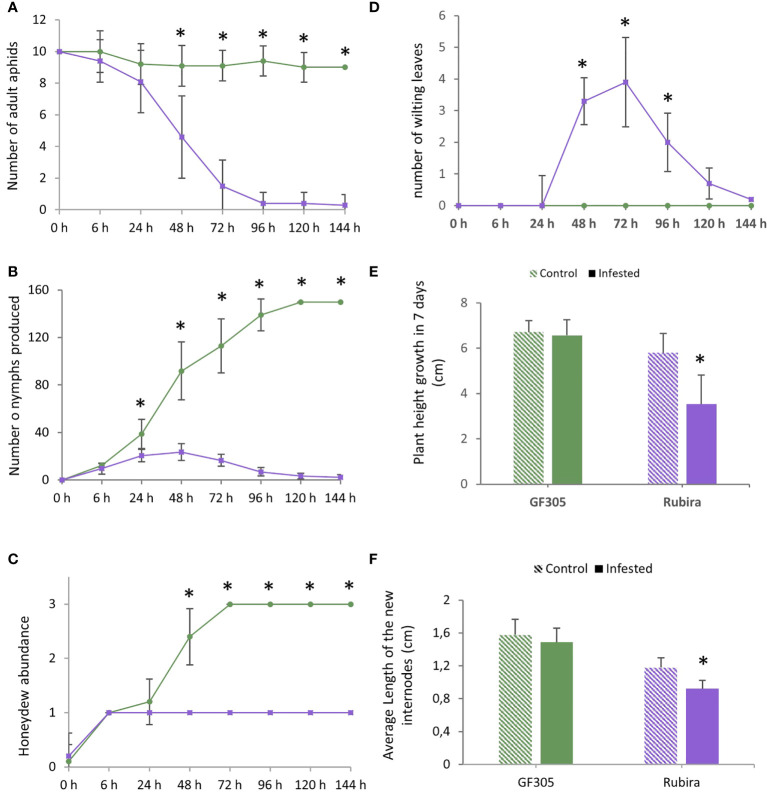
Aphid behavior and plant symptoms after infestation by GPA. Significant differences according to a Mann Whitney test are indicated (p.value<0.01 *). **(A)** Number of remaining aphids (out of ten adult deposited) on the susceptible GF305 (green circles) and the resistant Rubira (purple squares) seedlings over 6 days. **(B)** Number of nymphs produced. **(C)** Honeydew abundance expressed per classes (0: absence; 1: low; 2: medium; 3: high). **(D)** Number of wilting leaves on the growing shoot. **(E)** Height growth of the plants in seven days. **(F)** Average length of new internodes formed in 7 days (cm).

After infestation, the aphids deposited on the susceptible GF305 remained on the plant, with an average of nine aphids per plant, while they decreased rapidly on the resistant Rubira over 7 days, down to five aphids per plant 48 hpi, until all of them escaped 96 hpi (**Figure 1A**). The number of nymphs increased constantly on GF305 up to more than 150 (120 hpi), while after a moderate increase during 24 h on Rubira, it fell down to less than five 120 hpi (**Figure 1B**). The evolution of honeydew abundance on the leaves corroborates these trends: it was high on GF305 and low on Rubira, indicating an aphid feeding failure on the resistant plants (**Figure 1C**). GF305 plants also displayed typical symptoms of susceptibility 48 hpi, with young twisted leaves, whereas Rubira developed necrotic lesions on shoots and leaves and showed wilting leaves, (**Figure 1D** and **Supplementary Figure S1**). Seven dpi, infested Rubira plants had a 39% lower increase in stem height compared to control plants, while stem height increase of GF305 plants was not affected by infestation (**Figures 1E, F**).

### Overall trends in the transcriptomic and metabolomic responses to GPA *48 hpi*

**Figure 2:**
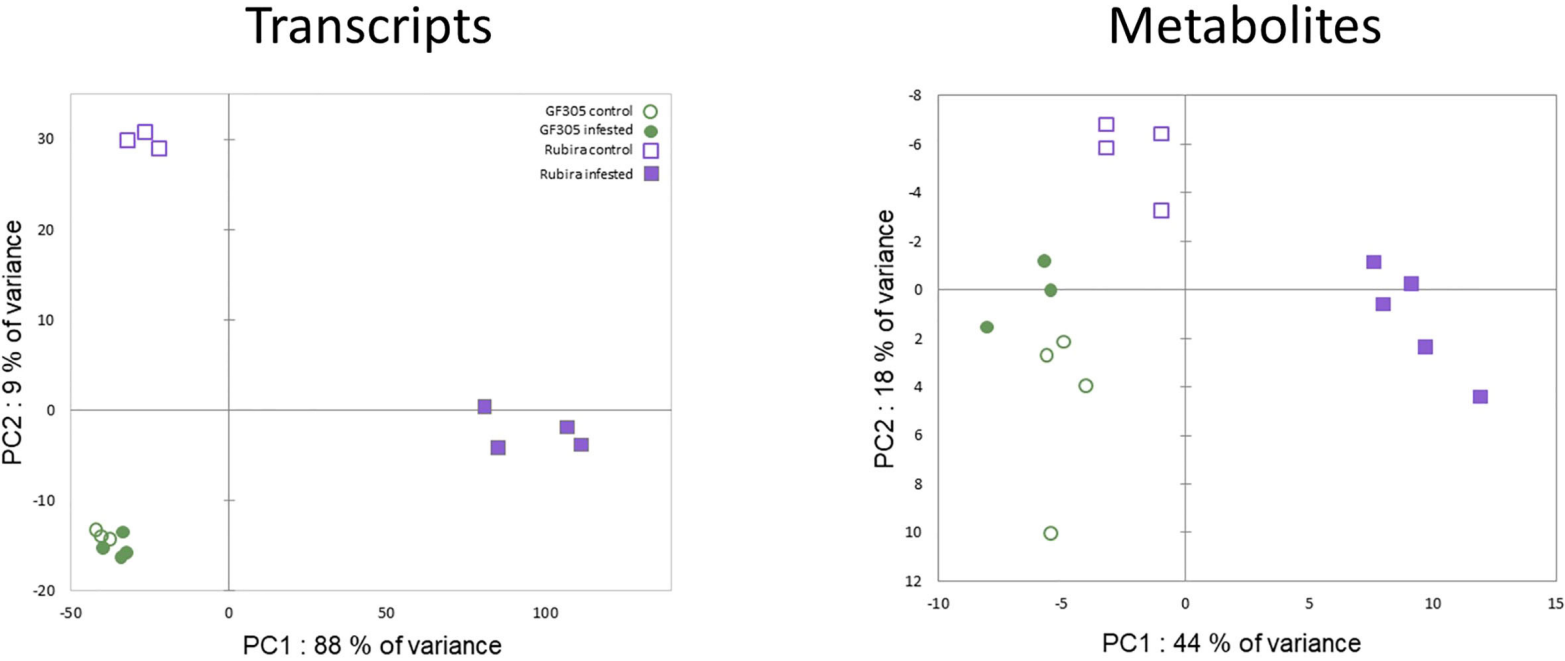
PCA score plots on raw transcription count data and metabolite semi-quantitative levels.

A PCA performed on the transcriptomic raw dataset (**Figure 2**) revealed that 88% of the total variance associated to PC1 was driven by aphid-induced differentially expressed genes (DEGs) in the resistant genotype Rubira. The second PC only expressed 9% of the total variance and was driven by the genotype differential gene expression. Among the 20 606 expressed genes, we found a total of 5743 DEGs: 284 genes were found differentially expressed between the 2 genotypes under control condition (175 DEGs upregulated in Rubira and 109 downregulated, **Supplementary Tables S1**, **S5**), only 35 DEGs were found between control and infested apices of the susceptible GF305 (34 upregulated and 1 downregulated, **Supplementary Table S6**) and 5424 DEGs between control and infested apices of the resistant Rubira (2990 upregulated and 2434 downregulated, **Supplementary Tables S1**, **S7** and **Table 1**).

**Table 1:** Selection of DEGs in the resistant genotype Rubira after GPA infestation, cited in the text and figures.

ID_Prunus	ID_Arabidopsis		% identity	Ortholog	LFC (RI/RC)	p.adj	Anotation_Arabidopsis *
**Ribosomal proteins**							
Prupe_7G102700		AT3G52580	91	Yes	-1.4	4.54E-02	RPS14C, 40S ribosomal protein S14-3
Prupe_6G300200		AT3G54210	73	Yes	-1.6	3.15E-06	RPL17; 50S ribosomal protein L17
Prupe_6G236800		AT2G37190	89	Yes	-1.4	8.91E-03	RPL12A; 60S ribosomal protein L12-1
**Receptor-like kinases and associated Serine/threonine-protein kinases**							
Prupe_4G029800		AT5G15730	46	No	8.3	2.30E-10	CRLK2; Calcium/calmodulin-regulated receptor-like kinase 2
Prupe_1G542300		AT1G09970	64	Yes	5.2	5.30E-116	RLK7; Receptor like protein kinase 7
Prupe_5G001000		AT2G33580	47	Yes	4.8	1.10E-15	LYK5; LysM-containing receptor-like kinase 5
Prupe_7G147500		AT2G33580	36	No	5.6	2.30E-03	LYK5; LysM-containing receptor-like kinase 5
Prupe_3G213100		AT3G21630	57	Yes	1.9	1.10E-08	CERK1; Chitin elicitor receptor kinase 1
Prupe_1G558900		AT4G33430	74	Yes	2.0	4.10E-15	BAK1; Brassinoid insensitive 1-associated receptor kinase 1
Prupe_8G115900		AT4G33950	87	Yes	1.3	9.10E-03	OST1; Open stomata 1
Prupe_1G437500		AT5G42750	48	Yes	1.8	2.30E-03	BKI1; BRI1 kinase inhibitor 1
Prupe_5G041700		AT5G46570	84	Yes	-1.7	6.90E-04	BSK2; Brassinosteroid-signaling kinase 2
Prupe_4G076500		AT5G46330	54	Yes	2.3	3.10E-10	FLS2; Flagellin sensitive 2
Prupe_3G099500		AT3G57750	40	No	8.7	3.36E-12	ZED1; HOPZ-ETI-deficient 1
Prupe_8G149300		AT3G57750	44	No	3.5	1.12E-14	ZED1; HOPZ-ETI-deficient 1
Prupe_8G149500		AT3G57750	50	No	7.6	1.61E-08	ZED1; HOPZ-ETI-deficient 1
Prupe_8G149400		AT3G57710	42	Yes	6.5	6.89E-53	RKS1; Resistance related kinase 1
Prupe_1G270700		AT1G14370	74	Yes	1.6	1.46E-05	PBL2; PBS1-like protein 2
**Wall-associated kinases**							
Prupe_4G093300		AT1G21270	46	Yes	7.9	1.25E-07	WAK2; Wall-associated receptor kinase 2
Prupe_5G171500		AT5G50290	64	Yes	-1.8	2.27E-03	Wall-associated receptor kinase galacturonan-binding protein
Prupe_7G145100		AT2G23450	55	Yes	2.0	7.28E-12	WAKL14; Wall-associated receptor kinase-like 14
Prupe_1G188400		AT1G16260	47	Yes	5.7	1.89E-02	WAKL8; Wall-associated receptor kinase-like 8
**Lectin-domain containing receptor kinase**							
Prupe_6G260000		AT2G37710	67	Yes	6.9	5.54E-25	LECRK41; L-type lectin-domain containing receptor kinase IV.1
**Nucleotide binding leucine-rich repeat domain proteins and associated helpers and transcription factors**							
Prupe_5G025300		AT3G07040	32.5	No	12.1	3.00E-02	RPM1; Disease resistance protein RPM1
Prupe_1G389500		AT4G33300	55	Yes	3.5	2.50E-41	ADR1-LIKE 1; Activated disease resistance 1 like 1
Prupe_8G199800		AT3G25070	43	Yes	1.9	5.50E-10	RIN4; RPM1-interacting protein 4
Prupe_7G198400		AT2G05940	72	Yes	3.5	1.40E-25	RIPK; RPM1-induced protein kinase
Prupe_7G139500		AT5G66900	42	Yes	3.8	5.40E-19	NRG1; N requirement gene 1; Probable disease resistance protein
Prupe_3G279300		AT3G52430	25	Yes	2.3	1.30E-22	SAG101; Senescence associated gene 101
Prupe_4G276500		AT3G52430	38	Yes	5.9	1.40E-46	PAD4; Lipase-like PAD5
Prupe_5G181000		AT3G48090	40	Yes	3.9	1.40E-47	EDS1; Enhanced disease susceptibility 1
Prupe_5G22360		AT1G73805	52	Yes	8.6	1.30E-88	SARD1; Protein SAR DEFICIENT 1
Prupe_6G315700		AT5G57580	37	No	3.4	2.60E-23	CBP60B; Calmodulin-binding protein 60 B
Prupe_4G036400		AT5G57580	62	No	1.5	1.95E-05	CBP60B; Calmodulin-binding protein 60 B
Prupe_7G160100		AT3G50950	61	Yes	5.6	4.93E-59	ZAR1; HOPZ-activated resistance 1
**MAP kinases**							
Prupe_1G564100		AT4G08500	69	No	1.3	4.30E-02	MEKK1; Mitogen-activated protein kinase kinase 1
Prupe_2G175200		AT4G01370	88	Yes	1.6	8.00E-07	MPK4; Mitogen-activated protein kinase 4
Prupe_6G091700		AT3G45640	86	Yes	3.4	8.00E-42	MPK3; Mitogen-activated protein kinase 3
Prupe_4G270800		AT1G53570	60	Yes	1.5	6.10E-06	MAPKKK3; Mitogen-activated protein kinase kinase kinase 3
**Hypersensitive response**							
Prupe_1G203300		AT4G28460	47	No	9.1	3.00E-11	PIP1; PAMP-induced secreted peptide 1
Prupe_6G228500		AT4G37290	40	Yes	9.2	2.40E-11	PIP2; PAMP-induced secreted peptide 2
Prupe_7G171200		AT4G35000	79	Yes	1.5	7.10E-05	APX3; Ascorbate peroxidase 3
Prupe_5G011300		AT4G35090	89	Yes	2.0	1.30E-06	CAT2; Catalase 2
Prupe_5G117000		AT4G23810	45	Yes	3.1	7.20E-12	WRKY53; Transcription factor WKRY53
Prupe_5G107400		AT1G64060	77	Yes	2.3	4.10E-12	RBOHF; Respiratory burst oxidase homolog protein F
Prupe_7G090800		AT3G10660	68	No	4.8	1.20E-21	CPK2; Calcium-dependent protein kinase 2
Prupe_7G064300		AT5G19450	79	No	2.3	1.70E-17	CPK8; Calcium-dependent protein kinase 8
Prupe_4G213800		AT3G20410	75	Yes	1.8	4.80E-08	CPK9; Calcium-dependent protein kinase 9
Prupe_4G233300		AT5G23580	44	No	5.5	1.60E-02	CPK12; Calcium-dependent protein kinase 12
Prupe_1G412900		AT4G33000	72	Yes	1.5	2.30E-02	CBL10; Calcineurin B-like protein 10
Prupe_2G195900		AT1G01140	76	Yes	1.8	1.00E-07	CIPK9; CBL-interacting serine/threonine-protein kinase 9
Prupe_5G186400		AT5G62740	65	Yes	2.6	6.63E-13	HIR1; HYPERSENSITIVE INDUCED REACTION 1
Prupe_1G268100		AT1G69840	93	Yes	1.7	2.69E-06	HIR2; HYPERSENSITIVE INDUCED REACTION 2
Prupe_2G281600		AT5G51570	87	Yes	1.4	6.33E-04	HIR4; HYPERSENSITIVE INDUCED REACTION 4
Prupe_7G158900		AT3G50930	65	Yes	2.6	1.26E-16	HSR4; HYPER-SENSITIVITY-RELATED 4
Prupe_7G097100		AT3G11660	62	Yes	1.5	4.54E-03	NHL1; NDR1/HIN1-LIKE 1
**Systemic acquired resistance/Salicylic acid/Pipecolic acid**							
Prupe_4G055900		AT4G18470	47	Yes	-2.2	9.76E-07	SNI1; SUPPRESSOR OF NPR1-1
Prupe_8G153800		AT2G14610	64	Yes	8.2	1.00E-80	PR-1; Pathogenesis related protein 1
Prupe_7G267900		AT2G43820	55	Yes	3.5	7.16E-24	SGT1; Salicylic acid glucosyltransferase 1
Prupe_7G142200		AT2G23620	60	Yes	-1.9	6.67E-05	MES1; METHYL ESTERASE 1
Prupe_6G168500		AT4G39460	80	Yes	-1.7	1.35E-08	SAMT1; S-ADENOSYLMETHIONINE TRANSPORTER 1
Prupe_4G107800		AT5G45110	62	No	1.6	2.66E-07	NPR3; NPR1-LIKE PROTEIN 3
Prupe_6G046900		AT2G38470	48	Yes	5.7	1.16E-63	WKRY33; Transcription factor WKRY33
Prupe_2G265000		AT3G56400	41	Yes	6.0	5.95E-65	WRKY70; Transcription factor WKRY70
Prupe_1G558600		AT2G13810	70	Yes	8.5	2.10E-11	ALD1; AGD2-like defense response protein 1
Prupe_2G302000		AT5G52810	66	Yes	2.1	1.10E-07	SARD4; SAR DEFICIENT 4
Prupe_7G193500		AT1G19250	70	Yes	9.7	2.20E-14	FMO1; Probable flavin-containing monooxygenase 1
Prupe_7G189800		AT4G33150	72	Yes	3.1	1.80E-10	LKR/SDH; Alpha-aminoadipic semialdehyde synthase
**Other Phytohormone pathways**							
Prupe_1G382900		AT1G75080	64	Yes	-1.6	2.30E-02	BZR1; Protein BRASSINAZOLE-RESISTANT 1
Prupe_7G264200		AT4G30610	74	Yes	-1.7	9.60E-03	BRS1; BRI1 suppressor
Prupe_1G505400		AT1G77760	78	Yes	2.4	4.60E-02	NR1; Nitrate reductase 1
**Autophagy**							
Prupe_4G215300		AT1G62040	91.453	Yes	1.6	6.65E-04	ATG8C; Autophagy-related protein 8c
Prupe_5G165900		AT4G24690	50.538	No	2.5	2.44E-05	NBR1; Neighbor of BRCA1
Prupe_8G193300		AT3G07370	66.545	Yes	1.4	2.33E-02	CHIP; Carboxyl terminus of HSC70-interacting protein
Prupe_7G106600		AT3G12580	61.047	No	6.8	5.60E-06	HSP70-4; Heat shock 70 kDa protein 4
**Polyols**							
Prupe_2G288800		AT5G51970	81	yes	1.6	1.40E-02	Sorbitol dehydrogenase
Prupe_8G101500		AT3G18830	65	No	2.3	3.30E-05	PLT5; Polyol transporter 5
**Glyoxylate metabolism**							
Prupe_4G258800		AT2G13360	86	Yes	2.2	5.60E-06	AGT1; Glyoxylate aminotransferase 1
Prupe_4G082600		AT3G14420	88	No	2.9	3.40E-20	GOX1; Glycolate oxidase
Prupe_3G048100		AT1G17650	77	Yes	-1.6	4.00E-04	GLYR2; Glyoxylate/succinic semialdehyde reductase 2, chloroplastic
Prupe_2G151800		AT1G12550	52	No	-2.2	6.20E-05	HPR3; Glyoxylate/hydroxypyruvate reductase
Prupe_3G219100		AT3G21720	84	Yes	4.3	2.50E-34	ICL; Isocitrate lyase
Prupe_4G216900		AT4G26910	70	Yes	-2.0	1.10E-03	ODH; Oxoglutarate dehydrogenase
Prupe_1G155800		AT5G27600	76	Yes	1.3	2.30E-02	LACS7; Long chain acyl-CoA synthetase 7, peroxisomal
Prupe_5G065100		AT4G16760	80	Yes	1.5	8.30E-04	ACX1; Peroxisomal acyl-coenzyme A oxidase 1
Prupe_6G181800		AT5G65110	83	Yes	1.9	1.90E-04	ACX2; Acyl-coenzyme A oxidase 2, peroxisomal
Prupe_1G003300		AT2G33150	86	Yes	1.6	3.90E-06	PED1; 3-ketoacyl-CoA thiolase 2, peroxisomal
Prupe_1G541200		AT4G37870	82	Yes	4.3	1.10E-28	PCK1; Phosphoenolpyruvate carboxykinase (ATP) 1
Prupe_4G170500		AT1G53240	85	Yes	-1.8	5.60E-09	MDH; malate dehydrogenase 1 mitochondriale
Prupe_4G058400		AT3G15020	82	Yes	-2.1	3.90E-06	MDH; malate dehydrogenase 2 mitochondriale
Prupe_4G116700		AT4G20070	71	Yes	2.1	2.60E-03	AAH; Allantoate deiminase
Prupe_4G045000		AT4G04955	73	Yes	3.3	3.20E-22	ALN; Allantoinase
Prupe_4G245700		AT4G34890	77	Yes	1.8	9.90E-09	XDH1; Xanthine dehydrogenase 1
**Glutamine and ammonium metabolisms**							
Prupe_2G269800		AT5G07440	89	Yes	2.1	1.30E-04	GDH2; Glutamate dehydrogenase 2
Prupe_2G311700		AT5G53460	83	Yes	2.5	3.50E-17	GLT1; Glutamate synthase 1
Prupe_6G054800		AT3G47340	85	Yes	4.5	1.10E-29	ASN1; Asparagine synthetase [glutamine-hydrolyzing] 1
Prupe_4G089000		AT4G21120	72	Yes	4.0	3.80E-33	CAT1; Cationic amino acid transporter 1
Prupe_3G211600		AT3G21670	74	Yes	1.4	3.70E-02	NPF6.4; Protein NRT1/PTR FAMILY 6.4
Prupe_1G052400		AT4G13510	80	Yes	1.8	2.10E-07	AMT1; Ammonium transporter 1
**Urea cycle/Proline metabolism**							
Prupe_1G463900		AT3G57560	70	Yes	-1.6	2.00E-03	NAGK; Acetylglutamate kinase
Prupe_8G115600		AT2G19940	81	Yes	-2.3	5.20E-10	NAGPR; N-acetyl-gamma-glutamyl-phosphate reductase
Prupe_6G093800		AT1G29900	82	Yes	-1.7	3.60E-07	CARB; Carbamoyl-phosphate synthase large chain, chloroplastic
Prupe_1G376500		AT1G75330	79	Yes	-1.8	1.60E-17	OTC; Ornithine carbamoyltransferase
Prupe_6G250400		AT2G37500	75	Yes	-1.4	5.30E-03	ArgJ; Arginine biosynthesis bifunctional protein ArgJ, chloroplastic
Prupe_5G153100		AT4G24830	79	Yes	-2.9	5.90E-19	ASSY; Arginosuccinate synthase
Prupe_3G092300		AT4G08900	30	No	2.2	8.70E-04	ARGAH1; Arginase 1
Prupe_2G076400		AT4G08870	68	No	6.8	3.50E-06	ARGAH2; Arginase 2
Prupe_5G019100		AT5G45380	79	Yes	2.0	5.20E-08	DUR3; Urea-proton symporter
Prupe_4G068700		AT4G18910	63	Yes	2.0	4.20E+64	NIP1; Aquaporin Nodulin-26-like major intrinsic protein
Prupe_5G138000		AT4G10380	82	Yes	2.0	4.70E-04	NIP5; Aquaporin Nodulin-26-like major intrinsic protein
Prupe_3G096600		AT1G80760	78	Yes	-1.9	1.60E-03	NIP6; Aquaporin Nodulin-26-like major intrinsic protein
Prupe_2G097000		AT4G35100	36	No	2.9	1.90E-03	PIP2; Aquaporin Plasma membrane intrinsic protein
Prupe_4G083300		AT5G46180	75	Yes	1.4	2.70E-02	DELTA-OAT; Ornithine aminotransferase, mitochondrial
Prupe_7G045400		AT5G14800	59	No	4.0	9.70E-17	PROC1; Pyrroline-5-carboxylate reductase
Prupe_3G243500		AT5G38710	60	Yes	2.1	1.10E-05	POX2; Proline dehydrogenase 2
Prupe_6G262300		AT3G55610	75	No	1.5	4.60E-03	P5CS2; Delta-1-pyrroline-5-carboxylate synthase
Prupe_5G187700		AT5G62530	81	Yes	1.5	9.20E-03	ALDH12A1; Delta-1-pyrroline-5-carboxylate dehydrogenase 12A1
**Methionine metabolism**							
Prupe_7G009200		AT5G17920	89	Yes	-1.6	1.90E-03	MS1; 5-methyltetrahydropteroyltriglutamate–homocysteine methyltransferase 1
Prupe_5G129800		AT1G64660	76	Yes	3.8	8.00E-35	MGL; Methionine gamma-lyase
**Leucine, isoleucine, valine biosynthesis**							
Prupe_6G116600		AT3G10050	68	No	-4.0	3.40E-02	OMR1; Threonine dehydratase
Prupe_5G043100		AT3G48560	80	Yes	-1.4	2.60E-02	ALS; Acetolactate synthase
Prupe_3G094300		AT1G80560	82	Yes	-1.5	4.30E-07	IMDH2; 3-isopropylmalate dehydrogenase 2
Prupe_1G003100		AT3G23940	82	Yes	-3.2	7.70E-21	DHAD; Dihydroxy-acid dehydratase
Prupe_1G416900		AT3G49680	59	No	3.0	1.70E-03	BCAT3; Branched-chain-amino-acid aminotransferase 3
**Lysine metabolism**							
Prupe_8G007000		AT1G31230	80	Yes	-2.7	8.60E-10	AKHSDH1;Aspartokinase/homoserine dehydrogenase 1
Prupe_1G334400		AT1G14810	81	Yes	-1.9	5.00E-05	ASADH; Aspartate-semialdehyde dehydrogenase
Prupe_6G152500		AT3G59890	81	Yes	-2.1	4.60E-13	DAPB2; 4-hydroxy-tetrahydrodipicolinate reductase 2
Prupe_8G156100		AT4G33680	81	No	-4.1	1.80E-19	AGD2; LL-diaminopimelate aminotransferase
Prupe_8G092300		AT5G11880	80	Yes	-1.5	1.90E-06	LYSA2; Diaminopimelate decarboxylase 2
Prupe_7G196700		AT1G54100	81	Yes	2.0	2.40E-12	ALDH7B4; Aldehyde dehydrogenase family 7 member B4
Prupe_7G189800		AT4G33150	72	Yes	3.1	1.80E-10	LKR/SDH; Alpha-aminoadipic semialdehyde synthase
**Beta-alanine and Beta-aminosiobutyrate biosynthesis**							
Prupe_4G264100		AT5G12200	81	No	1.8	8.40E-12	PYD2; Dihydropyrimidinase
Prupe_8G070200		AT5G64370	84	Yes	2.0	1.30E-08	PYD3; Beta-ureidopropionase
**Shikimate pathway**							
Prupe_2G143700		AT2G45300	77	Yes	-1.8	2.50E-07	EPSPS; 3-phosphoshikimate 1-carboxyvinyltransferase
Prupe_1G393400		AT5G10870	67	Yes	2.2	4.30E-09	CM2; Chorismate mutase 2
Prupe_1G281400		AT1G69370	66	Yes	-3.2	8.40E-23	CM3; Chorismate mutase 3
Prupe_6G119200		AT1G08250	80	Yes	-1.4	3.60E-02	PDT6; Arogenate dehydratase/prephenate dehydratase 6
**Phenylpropanoid and flavonoïds biosynthesis**							
Prupe_6G040400		AT2G30490	86	Yes	-1.6	1.20E-04	CYP73A5; Trans-cinnamate 4-monooxygenase
Prupe_2G326300		AT1G65060	70	No	-1.9	1.20E-04	4CL3; 4-coumarate-CoA ligase 3
Prupe_3G101400		AT5G48930	68	No	-3.8	8.60E-06	HCT; Shikimate O-hydroxycinnamoyltransferase
Prupe_1G580300		AT2G40890	80	No	-2.1	4.20E-06	CYP98A3; C3’H; p-coumaroylshikimate/quinate 3’-hydrolxylase
Prupe_8G135300		AT3G19450	78	Yes	1.6	2.40E-06	CAD4; Cinnamyl alcohol dehydrogenase 4
Prupe_6G207700		AT4G37980	75	No	9.1	1.10E-11	CAD7; Cinnamyl alcohol dehydrogenase 7
Prupe_1G003000		AT5G13930	85	Yes	-2.0	6.80E-06	CHS; Chalcone synthase
Prupe_2G225200		AT3G55120	71	Yes	-2.6	7.30E-29	Chalcone isomerase
Prupe_1G376400		AT5G42800	70	Yes	-2.9	2.50E-18	DFR; Dihydroflavonol 4-reductase
Prupe_7G168300		AT3G51240	81	Yes	-1.9	1.60E-11	F3H; Naringenin,2-oxoglutarate 3-dioxygenase
Prupe_1G502700		AT5G08640	61	Yes	-4.2	1.80E-02	FLS; Flavonol synthase
Prupe_2G199600		AT5G54160	56	No	-3.1	3.50E-08	OMT1; Flavone 3’-O-methyltransferase 1

Significant variations (p.adj) were determined using the DeSEQ2 package (R) at the threshold α = 0.05. Repressed genes with negative Log fold changes (LFC) are indicated in blue and up-regulated genes are indicated in red. (*) https://www.uniprot.org

**Figure 3:**
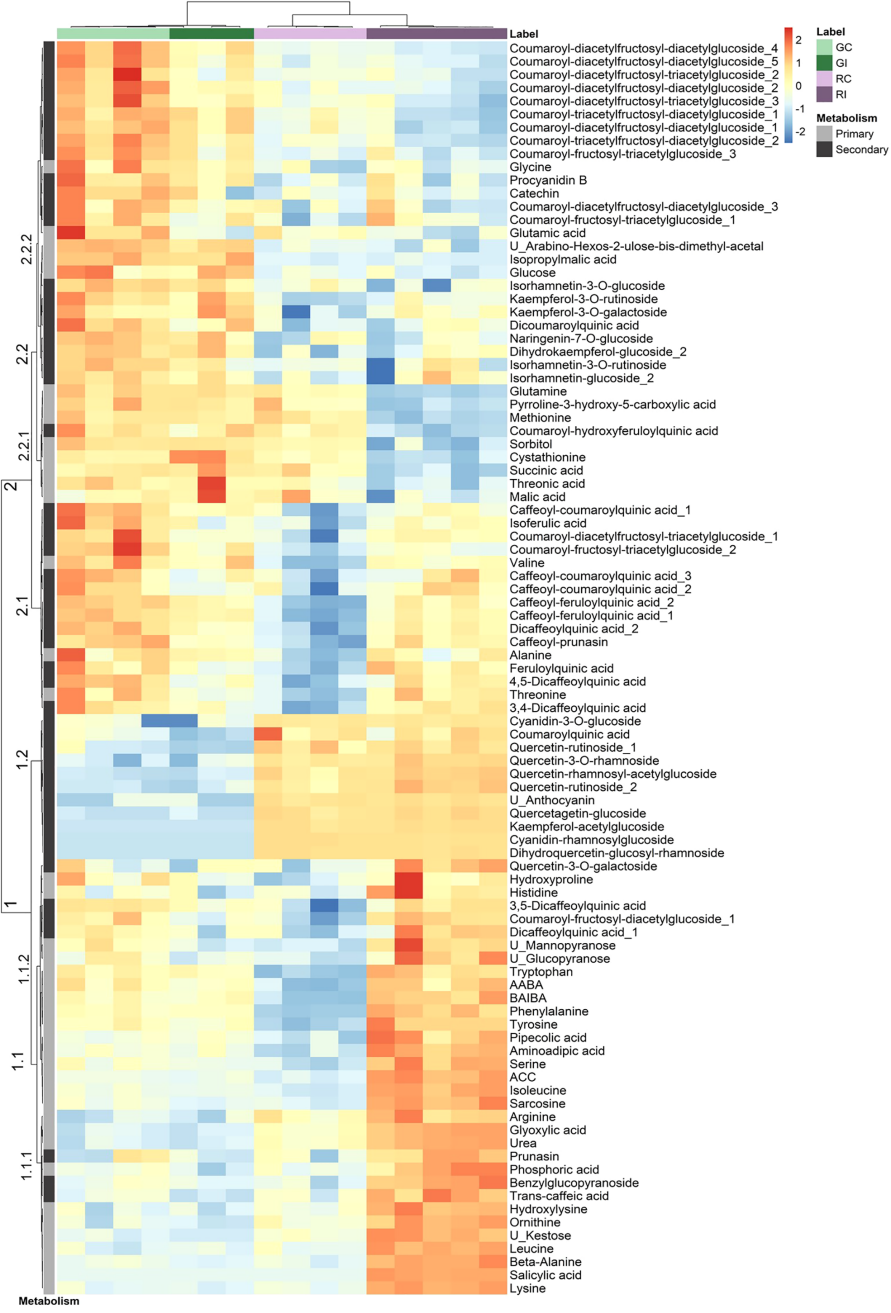
Hierarchical clustering analysis of a subset of metabolites displaying a significant difference (Kruskall-Wallis, p<0.05) between uninfested GF305 and Rubira control plants (GC vs RC) or between control and GPA-infested plants (Gc vs GI and RC vs RI) 48 hpi. HCA was performed using the Euclidian distance and the Ward algorithm on data after log transformation and Pareto normalization (mean-centered and divided by the square root of the standard deviation). Primary and secondary metabolites are indicated by a gray scale on the left. GC, GF305 control; GI, GF305 infested; RC, Rubira control; RI, Rubira infested.

A combination of targeted and non-targeted metabolomic approaches provided the relative contents of 138 metabolites, including 3 defense phytohormones, 69 primary and 66 secondary compounds. The reliability of identification was determined as recommended by Schymanski et al. (2014): 69 compounds were identified at level 1, 22 at level 2 and 47 at level 3. The distribution of these compounds in structural families is shown in the **Supplementary Figure S2**. A total of 94 compounds showed significant variation in at least one condition: 73 discriminated the uninfested genotypes (**Supplementary Table S8**), 12 changed after infestation in GF305 (**Supplementary Table S9**) and 54 in Rubira (**Supplementary Table S10**). Interestingly, the trends of metabolomics data revealed by a PCA was similar to that observed for gene expression (**Figure 2**): PC1 explained 44% of the variance and discriminated aphid-infested Rubira samples from other samples, while PC2 explained only 18% of the variance and was associated to the discrimination of the uninfested genotypes, confirming that the molecular response of Rubira to GPA was several orders of magnitude greater than that of GF305 and also exceeded the constitutive difference between the genotypes. A hierarchical clustering analysis conducted on the 94 discriminant compounds revealed clusters of metabolites discriminating the genotypes and/or responding to infestation (**Figure 3**). The clusters 2.2.2 and 1.2 gather mostly secondary metabolites remaining stable after infestation but with higher constitutive content in GF305 and Rubira respectively. The cluster 2.2.2 is a mixture of flavonoids and coumaric acid derivatives while the cluster 1.2 contains mainly flavonols and anthocyanins. The cluster 2.1 gathers mainly caffeic acid esters that were constitutively more abundant in GF305 but decreased in this genotype after infestation while they increased in Rubira. Finally, the clusters 1.1 and 2.2.1 show respectively the specific aphid-induced accumulation and depletion of primary metabolites in Rubira.

### Constitutive differences between the susceptible and resistant genotypes

**Figure 4:**
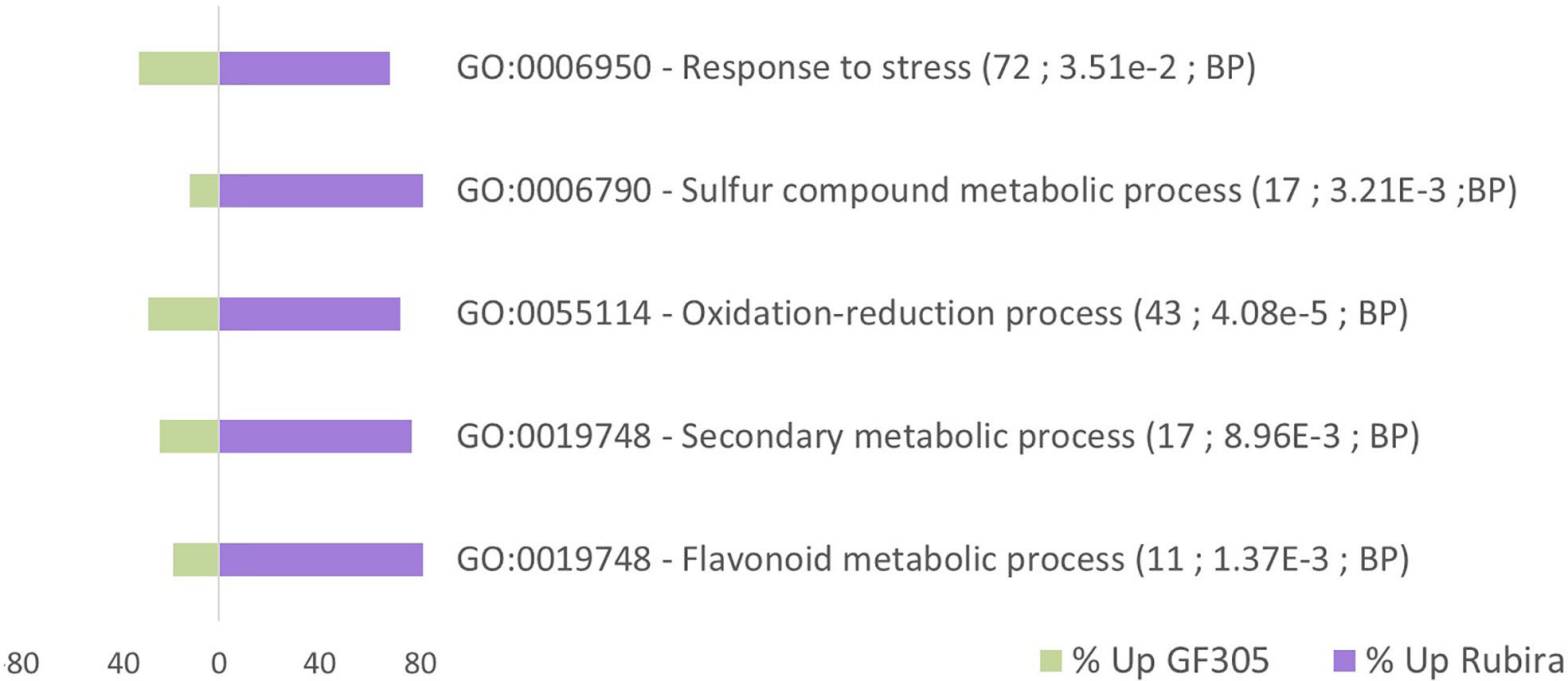
Gene Ontology (GO) enrichment analysis for the comparison of constitutive gene expression in the uninfested resistant Rubira compared to the susceptible GF305. Bars indicate the percentage of upregulated genes associated to GO terms. A total of 217 markers were mapped in this enrichment analysis. GO terms with at least a 1.5-fold over-representation and an enrichment adjusted p.value lower than 0.05 are presented. For each GO term, details are given in parentheses: (i) number of P. persica GO annotated genes, (ii) adjusted p.value of enrichment, (iii) GO groups (BP: Biological process).

The Gene Ontology (GO) enrichment analysis of the 284 DEGs highlighted five functions, most upregulated genes being found in Rubira (**Figure 4**; **Supplementary Table S5**). The greater differences relate to flavonoid metabolism (GO:0019748, GO:0009812), with most genes globally induced in Rubira. This is consistent with the accumulation of quercetin-3-O-rhamnoside and especially cyanidin-3-O-glucoside (**Supplementary Figure S3**), responsible for the red colour of Rubira’s foliage. This trait is controlled by the *Gr* locus which is independent of aphid resistance (Pascal et al., 2002) and located on chromosome 6 (Lambert and Pascal, 2011). Indeed, *PpMYB10.4* (*Prupe_6G175900*), a *Gr* gene candidate encoding a MYB transcription factor (Zhou et al., 2014b) was highly induced in Rubira. The high anthocyanin content is further explained by the strong expression of the orthologs of leucoanthocyanidin dioxygenase (*Prupe_5G086700*) and anthocyanidin-3-O-glucosyltransferase (*Prupe_2G324700*), involved in the anthocyanin biosynthesis pathway, and *PpGST1* (*Prupe_3G013600*), a glutathione S-transferase, orthologous to *GSTF12* and involved in the transport of anthocyanins essential for peach coloration (Zhao et al., 2020). In contrast, GF305 had higher levels of isorhamnetin-3-O-glucoside, isorhamnetin-3-O-rutinoside and kaempferol-3-O-rutinoside, as well as caffeoylquinic acids, phenylpropanoid-acetyl-sucrose esters and the three aromatic amino acids, phenylalanine, tyrosine and tryptophan.

### Responses to infestation in the susceptible genotype

Only 35 genes were differentially expressed in GF305 after infestation, all but one induced (**Supplementary Table S6**). Of the 34 genes induced, 27 were also induced in Rubira after infestation. The small number of genes involved did not allow for an enrichment study but many genes could be *a priori* assigned to a few functional groups. The most remarkable DEG, *Prupe_5G025300*, is a *NLR* gene that shares homology (32.5% identity) with its Blastp best hit in Arabidopsis, the resistance protein *RPM1* (Gao et al., 2011). It was the only gene repressed in GF305 after infestation while it was induced in Rubira. Among the genes that were induced, several are involved in brassinosteroid (BR) regulation, like *Prupe_1G520800*, a close homolog of *EXORDIUM*, which is a central coordinator of BR-dependent growth control (Coll-Garcia et al., 2004), also involved in response to herbivory (Mohanta et al., 2012), and *Prupe_5G222200*, ortholog of the basic helix-loop-helix (bHLH) transcription factor BEE3, a positive and early regulator of BR signalling (Friedrichsen et al., 2002). Several DEGs are homologs of Arabidopsis genes regulated by BRs and also regulated by auxin, like *KRP1* (Gupta et al., 2015), coding for a Calcium binding EF-hand family protein linked to calcium homeostasis during the photoperiod and possibly controlling the diurnal sucrose synthase activity (Solomon et al., 2010). Finally, only twelve metabolites showed statistically significant variations after infestation (**Supplementary Table S9**). All of them decreased: glutamate, threonine and nine secondary metabolites related to defense, including caffeoyl-prunasin, caffeoylquinic and dicaffeoylquinic acids and coumaroyl-acetyl-sucrose esters (**Supplementary Figure S4**).

### Responses to infestation in the resistant genotype

**Figure 5:**
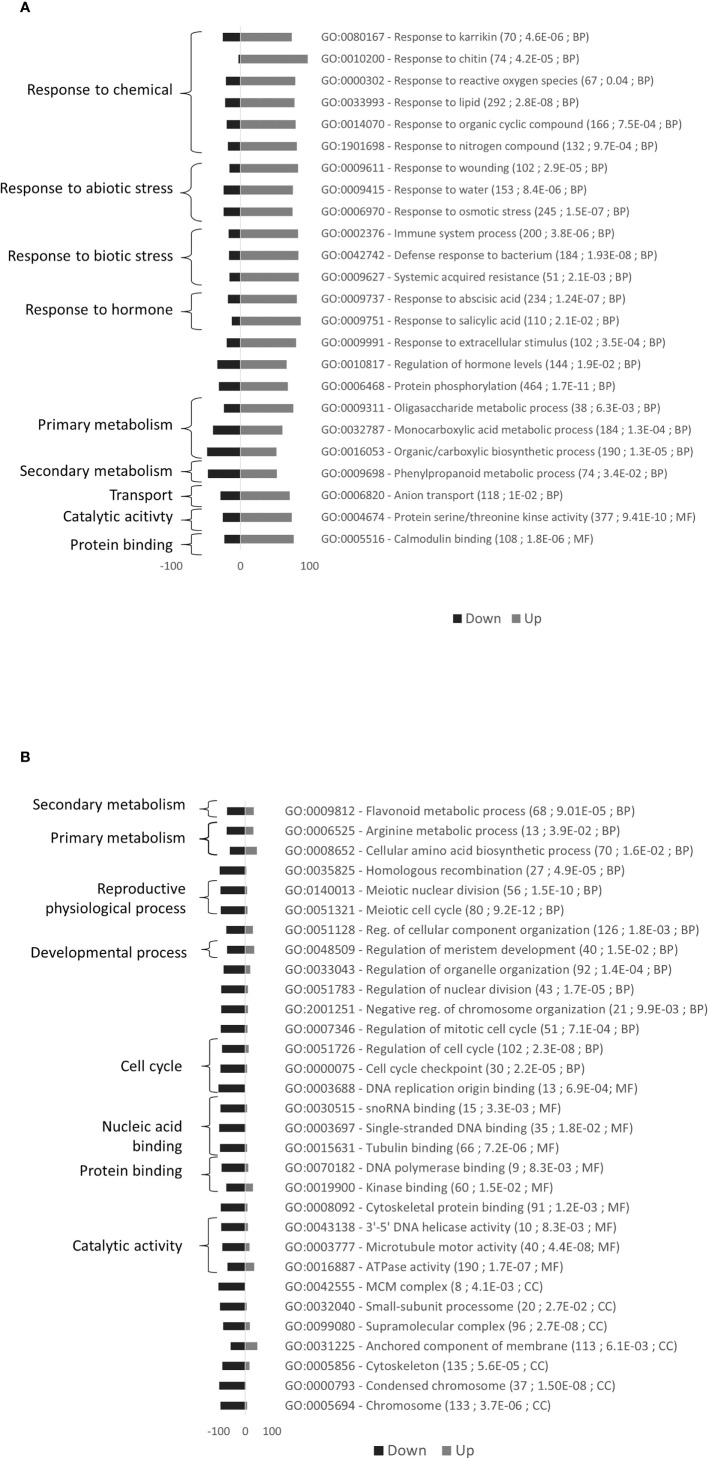
Gene Ontology (GO) enrichment analysis for the comparison of gene expression variations induced by GPA infestation of Rubira. **(A)** Upregulated genes. **(B)** Downregulated genes. Bars indicate the percentage of genes up- or down-regulated associated to GO terms. A total of 3376 markers were mapped in this enrichment analysis. GO terms with at least a 1.5-fold over-representation and an enrichment adjusted p.value lower than 0.05 are presented. For each GO term, details are given in parentheses: (i) number of *P. persica* GO annotated genes, (ii) adjusted p.value of enrichment, (iii) GO groups (BP, Biological process; CC, Cellular compartment; MF, Molecular Function).

24 GO terms including stress and metabolism were mostly upregulated and 31 GO terms including cell division were mostly downregulated in Rubira after infestation (**Figure 5**). DEGs commented below are exposed in the **Table 1**, the full annotation is presented in the **Supplementary Table S7**.

#### Repression of cell division and growth

Most downregulated genes are operating in the many processes of cell division and growth (**Figure 5**), including supramolecular complex (GO:0099080), chromosomes (GO:0005694) and protein complexes such as the mini-chromosome maintenance (MCM) complex (GO:0042555), a protein complex necessary for the initiation and regulation of DNA replication (Tuteja et al., 2011), or the small-subunit processome (GO:0032040), a preribosomal complex required for the 18S ribosomal RNA biogenesis (Bernstein et al., 2004). The molecular functions involved concern interaction with nucleic acids for DNA replication, RNA transcription, and cell division, such as DNA polymerase binding (GO:0070182) and single-stranded DNA binding (GO:0003697). The downregulated genes are also associated to the regulation of cell cycle (GO:0051726) and meristem development (GO:0048507). Accordingly, nearly sixty genes coding for ribosomal proteins were downregulated, such as *Prupe_7G102700*, *Prupe_6G300200*, and *Prupe_6G236800*, the respective orthologs of *RPS14C*, *RPL17* and *RPL12A* (**Table 1**).

#### Induced expression of immune receptor genes

The genes associated with protein serine/threonine kinase activity (GO:0004674) and calmodulin binding (GO:0005516) were mostly upregulated (**Figure 5**). Biotic stress is first perceived by transmembrane protein kinases, such as RLKs, comprising an extracellular domain that perceives stimuli and an intracellular domain transmitting information to several cytoplasmic protein kinases subfamilies: mitogen-activated protein kinases (MAPKs), calcium-dependent protein kinases (CPKs) and calcineurin B-like proteins (CBLs) (Kurusu et al., 2010; Tang et al., 2017). Many genes coding for these proteins were upregulated in Rubira after infestation, probably involving reactive oxygen species (ROS)-mediated signal transduction (response to reactive oxygen species, GO:0000302).

Homologs of typical PRR genes involved in PTI were activated (**Table 1**), like *Prupe_4G029800*, an homolog of *CLRK1*/*CLRK2* in Arabidopsis and homolog of *Csa5M642150* in cucumber, identified as a candidate for resistance to *Aphis gossypii* (Liang et al., 2016). *Prupe_1G542300* is the ortholog of *RLK7*, a cell surface receptor which triggers immune response upon detection of the PAMP-induced secreted peptide 1 (PIP1) and controls the accumulation of ROS detoxifying enzymes such as the superoxide dismutase (Pitorre et al., 2010; Hou et al., 2014). *Prupe_3G213100* and *Prupe_5G001000*, orthologs of chitin elicitor receptor kinase 1 (*CERK1*) and Lysin motif-containing receptor-like kinase 5 (*LYK5*) respectively, both involved in chitin detection (Erwig et al., 2017), were up-regulated as well. *Prupe_1G558900* encoding a homolog of the co-receptor *BAK1*, involved in brassinosteroid signalling and PTI, through its association with BRI1 or FLS2 respectively (Li et al., 2002; Chinchilla et al., 2007) was upregulated, as well as *BKI1* (Wang and Chory, 2006) and homologs of *FLS2* (Gómez-Gómez and Boller, 2000). BAK1 also associates to OST1 (*Prupe_8G115900*, ortholog), a ROS/ABA dependent activator of RBOHF (Mittler and Blumwald, 2015). On the contrary *Prupe_5G041700*, ortholog of *BSK2* involved in BR signalling downstream of BRI1, was downregulated (Tang et al., 2008). It is also worth mentioning the impact of infestation of Rubira on the expression of genes encoding *WAKs* (Wall-associated kinases, like *Prupe_4G093300*, the ortholog of *WAK2*) and *WAKLs* (WAK-like, such as *Prupe_7G145100*, the ortholog of *WAKL14*), with 14 out of 15 homologs being induced and only one repressed.

Many NLR genes were also up-regulated, like thirteen homologs of RPM1 and the orthologs of functionally associated defense genes *Prupe_8G19980* (*RIN4*) and *Prupe_7G198400 (RIPK)*. *Prupe_1G389500*, the ortholog of *ADR1-like1*, and *NRG1* (**Table 1**) are CNLs, working downstream in parallel branches as helpers of TNLs necessary for TNL-mediated immunity (Wu et al., 2019). These proteins associate with nucleocytoplasmic lipase-like proteins, like EDS1 and PAD4 or SAG101, that enhance TNL responses (Lapin et al., 2019; Wu et al., 2019). The orthologs of *EDS1* (*Prupe_5G181000*), *PAD4* (*Prupe_4G276500*) and *SAG101* (*Prupe_3G279300*) were all upregulated in infested Rubira (**Table 1**). In addition, *Prupe_7G160100*, the ortholog of the CNL *ZAR1*, was also induced, as were homologs of the associated RLCKs: *ZED1*, *RKS1* (*Prupe_8G149400*, orthologs) and *PBL2* (*Prupe_1G270700*, ortholog) (Duxbury et al., 2021).

#### Upregulation of ROS signalling, phosphorylation cascades and HR

Downstream detection systems, defense signal transduction mechanisms were activated in Rubira. Upregulated MAPK phosphorylation cascades included *Prupe_1G564100* an homolog of the H_2_O_2_ inducible gene *MEKK1*, which in turn induced *MPK4* (*Prupe_2G175200*, ortholog) expression (Pitzschke et al., 2009) (**Table 1**). These two central regulators of redox homeostasis in plants control detoxification enzymes, such as the catalase *CAT2* (*Prupe_5G011300*, ortholog) and can induce expression of *WRKY53* (*Prupe_5G117000*, ortholog) (Pitzschke et al., 2009), a transcription factor responding to infection or abiotic stress (Hu et al., 2012). *Prupe_6G091700*, encoding the ortholog of the important pathogen-responsive MPK3 (Lang et al., 2022) was also upregulated. Other peach genes encoding protein kinases subfamilies were strongly represented and generally induced: *CPK* (*e.g. Prupe_4G213800*, ortholog of *CPK9*), *CBL* (*e.g. Prupe_1G412900*, ortholog of *CBL10*) and CBL-interacting protein kinases (*CIPKs*, (*e.g. Prupe_2G195900*, ortholog of *CIPK9*). These proteins share a Ca^2+^ activated EF-hand motif and a kinase domain triggering phosphorylation events (Marcec et al., 2019). CPKs can activate respiratory burst oxidases (RBOH), such as RBOHF (*Prupe_5G107400*, ortholog), a family of NADPH-oxidase producing ${\text{O}}_{2}^{-}$ in the apoplasm, which turns into H_2_O_2_ (Kimura et al., 2017).

The presence of necrotic spots on Rubira near the secondary veins and on the stems after GPA infestation suggests the establishment of a hypersensitive response (HR). This was associated to the transcriptional upregulation of genes involved in response to reactive oxygen species (GO:0000302) and protein phosphorylation (GO:0006468), which are rapid events generally preceding HR. Orthologs of important HR genes were upregulated, like the orthologs of *HIR1*, *HIR2* and *HIR4* encoding hypersensitive-induced reaction (HIR) proteins, members of the Proliferation, Ion and Death superfamily and involved in the development of spontaneous lesions (Choi et al., 2011; Qi et al., 2011). Other induced HR markers were *Prupe_7G158900*, the ortholog of *HSR4* (Zhang et al., 2014)*, Prupe_7G097100* the ortholog of *NHL1*, a member of NDR1/HIN1-like (NHL) gene family including *NDR1* in Arabidopsis (Knepper et al., 2011), required for HR and for resistance conferred by R genes, and *HIN1*, initially identified in tobacco as an HR marker based on its induction by harpins (Pontier et al., 1999).

#### Changes in phytohormone pathways

In aphid-infested Rubira, 84% of the DEGs classified in SAR (GO:0009627) were upregulated (**Figure 5**), including the two main homologs of the transcription factor *WRKY33* (the ortholog *Prupe_6G046900* and *Prupe_6G286000*), a global regulator of SAR (Wang et al., 2018a; Barco and Clay, 2020) and the two main homologs of *EDS1* (the ortholog *Prupe_5G181000* and *Prupe_5G180900*), a positive regulator of HR and SAR through SA signalling (Gao et al., 2015; Dongus and Parker, 2021). Moreover, one of the nine repressed genes in this GO category was *SNI1*, which encodes a core protein capable of suppressing the expression of PRs and more generally of SAR (Li et al., 1999). SAR is controlled by two immune pathways, one involves SA and PR proteins, the other pipecolic acid. The activation of the SA pathway was demonstrated by accumulation of SA and SAG in Rubira 48 hpi (**Figure 3**, cluster 1.1., **Supplementary Table S10**, **Supplementary Figure S5**) as well as the induction of 87% of SA response genes (GO:0009751) and induction of the ortholog (*Prupe_8G153800*) and three homologs of *PR-1*. The transcription of many genes implicated in SA regulation was impacted (**Table 1**), like *Prupe_7G267900*, the ortholog of *SGT1* coding for a glycosyltransferase catalyzing the formation of SAG and SA glucose ester (SGE), *Prupe_7G142200* and *Prupe_6G168500*, the respective orthologs of *MES1* and *SAMT1* involved in the production of methyl-salicylate (MeSA). Upstream, the ortholog of a gene encoding a transcription factor recruited in the promoter of many SA and PR biosynthesis genes, *SARD1* (*Prupe_5G22360*), was upregulated (Sun et al., 2015), as well as homologs of *CBP60b*, that positively regulates immunity genes (Li et al., 2021), including *SARD1* (Huang et al., 2021). The ortholog of *WRKY70* (*Prupe_2G265000*) was up-regulated as well. This transcription factor regulating the balance between SA and JA signalling defense pathways (Li et al., 2006) is required for the resistance to aphids and nematodes mediated by *Mi-1* in tomato and was found to be upregulated by SA and downregulated by JA (Atamian et al., 2012; Chen et al., 2021). Interestingly the ortholog of the *MYB44* transcription factor *Prupe_1G430000* was also induced. *MYB44* is a transcriptional activator of *WRKY70* expression that activates SA-mediated defenses and represses JA-mediated defenses (Shim et al., 2013). MYB44 plays a critical role in resistance to GPA in *Arabidopsis* (Lü et al., 2013) and is highly expressed in response to *Sitobion avenae* and associated to phloem-based defenses in wheat (Zhai et al., 2017).

The second SAR pathway depends on the accumulation of *N*-hydroxypipecolic acid (NHP) (Chen et al., 2018; Hartmann and Zeier, 2018) and its precursor pipecolic acid (Návarová et al., 2012). NHP could not be detected in this study but its direct precursor, pipecolic acid, was found to accumulate 48 hpi in Rubira (**Supplementary Figure S5**). Orthologs of NPH biosynthetic pathway genes were all upregulated: *ALD1* (*Prupe_1G558600*), coding for an aminotransferase that catalyzes the first step of lysine catabolism to ϵ-amino-α-keto caproic acid (Návarová et al., 2012; Ding et al., 2016; Hartmann et al., 2017), *SARD4* (*Prupe_2G302000*), which allows the formation of pipecolate from Δ1-piperidine-2-carboxylate (Ding et al., 2016) and *FMO1* (*Prupe_7G193500*) allowing the hydroxylation of pipecolate to NHP (Hartmann et al., 2018).

Other phytohormone pathways were regulated as well upon infestation: orthologs of *BZR1* (*Prupe_1G382900*), a positive regulator of BR signalling pathways and *BRS1* (*Prupe_7G264200*), a carboxypeptidase involved in BR signalling, were repressed after infestation (**Table 1**). On the contrary, NO signalling was probably activated, with the upregulation of the Nitrate reductase 1 ortholog (NR1, Prupe_1G505400).

#### Upregulation of autophagy

Several genes involved in autophagy were upregulated upon infestation (**Table 1**). Autophagy-related 8 genes (*e.g. Prupe_4G215300*, an *ATG8c* isoform) produce proteins that covalently attach to autophagic membranes and can bind autophagy receptors such as NBR1 (*Prupe_5G165900*, homolog) to deliver cargo in vacuole for degradation (Kirkin et al., 2009; Yoshimoto and Ohsumi, 2018). Other markers of autophagy include *Prupe_8G193300*, the ortholog of *CHIP*, coding for a ubiquitin ligase and *Prupe_7G106600*, the homolog of a heat shock protein HSC70-4 gene, which both mediate cytosolic protein aggregate degradation (Lee et al., 2009; Zhou et al., 2014a).

#### Reconfiguration of central metabolism

A handful of metabolites and genes indicate that the central carbon metabolism was affected by infestation in Rubira (**Table 1**; **Supplementary Table S10**). Sorbitol level decreased massively 48 hpi (**Figure 3**, cluster 2.2.1. and **Supplementary Figure S6A**), while the upregulation of homologs of polyol transporters and *Prupe_2G288800*, the ortholog of sorbitol dehydrogenase converting sorbitol to fructose (Nosarzewski et al., 2012), suggest an active depletion of the sorbitol pool in infested apices. On the contrary, glyoxylate accumulated 48 hpi (**Figure 3**, cluster 1.1., **Supplementary Figure S7A**) and several DEGs indicate that the three pathways contributing to glyoxylate biosynthesis, *i.e.* photorespiration, the glyoxylate shunt, and purine catabolism, were all activated. The photorespiratory production of glyoxylate (Dellero et al., 2016) is revealed by several cues: (i) the accumulation of serine, (ii) the induction of anabolic reactions genes: the ortholog of serine-glyoxylate aminotransferase 1 (*AGT1*, *Prupe_4G258800*) and homolog of glyoxylate oxidase (*GOX1*) and (iii) the repression of catabolic reactions: *Prupe_3G048100*, the ortholog of the Glyoxylate/succinic semialdehyde reductase 2 (*GLYR2*) and a homolog of Glyoxylate/hydroxypyruvate reductase (*HPR3*) (**Table 1**). A key enzyme of the glyoxylate shunt produces glyoxylate and succinate from isocitrate: the isocitrate lyase (ICL), whose ortholog gene, *Prupe_3G219100*, was also induced in Rubira 48 hpi. This anaplerotic pathway shunts the tricarboxylic acid cycle and indeed, orthologs of the TCA cycle *ODH* (*Prupe_4G216900*) and *MDH* (*Prupe_4G170500*) were downregulated after infestation of Rubira. Consistently, citrate and glyoxylate increased and succinate and malate pools were reduced, supporting the idea of a downregulation of the TCA cycle in favor of the glyoxylate shunt. The shunt is fed by fatty acid-derived acetyl-CoA, condensed onto oxaloacetate to produce citrate (Smith, 2002). In our data, the induction of orthologs of the long chain acyl-CoA synthase 7 (*LACS7*, *Prupe_1G155800*), acyl-CoA oxidases (*ACX1 Prupe_5G065100*, *ACX2, Prupe_6G181800)* and 3-ketoacyl-CoA thiolase 2 (*PED1*, *Prupe_1G003300*), traduced the activation of lipid beta-oxidation. Moreover, the strong upregulation of *Prupe_1G541200*, the ortholog of *PCK1*, revealed an activation of gluconeogenesis, a primary function of the glyoxylate shunt (Smith, 2002). The third pathway of glyoxylate production involves the degradation of purine (Werner and Witte, 2011), attested here by the induction of orthologs of allantoate deaminase (*AAH*, *Prupe_4G116700*), allantoinase (*ALN*, *Prupe_4G045000*) and xanthine dehydrogenase 1 (*XDH1*, *Prupe_4G245700*) (**Table 1**), the latter producing glyoxylate and urea, which both accumulated 48 hpi (**Supplementary Figure S7A, C**).

The reconfiguration of central N metabolism involved genes in the categories Arginine metabolic process (GO: 0006525) and Cellular amino acid biosynthesis process (GO: 0008652), mostly downregulated (**Figure 5**). Indeed, in infested Rubira, the major amino acids involved in nitrogen assimilation and distribution were either stable, for aspartate and asparagine, or reduced for glutamate and glutamine (**Supplementary Figure S6B**, **S7B**). Decrease in glutamate could be related to the upregulation of *GDH2* ortholog (*Prupe_2G269800*), and the decrease in glutamine could be linked to the upregulation of *GLT1* ortholog (*Prupe_2G311700*) and glutamine hydrolyzing asparagine synthetase ortholog (*ASN1*, *Prupe_6G054800*) (**Table 1**). The strong induction of *ASN1* after infestation, while the asparagine pool remained constant, suggests the activation of a mechanism exporting nitrogen out of the infested apices. This hypothesis is supported by the upregulation of homologs of cationic amino acid transporter genes (*CATs*) and nitrate or ammonium transporter genes (*NPF*, *AMT1*). Nitrogen metabolism was also impacted at the level of the urea cycle, a pathway producing urea from arginine and recycling ornithine in the process (Winter et al., 2015). Citrulline decreased 48 hpi but ornithine and arginine accumulated (**Supplementary Figure S6C**, **S7C**). Paradoxically, the upstream pathways participating in arginine biosynthesis were transcriptionally downregulated: *NAGK*, *CARB*, *OCT*, *ArgJ* and *ASSY* orthologs, whereas *ARGAH* homologs, involved in the last step turning arginine into ornithine and urea, were upregulated. Urea content actually increased, concomitantly with the induction of the *DUR3* ortholog *Prupe_5G019100*, encoding an active urea transporter (Bohner et al., 2015) as well as some aquaporin orthologs (*NIPs*) allowing the passive transport of this molecule (Matiz et al., 2019). The catabolism of ornithine was also activated toward the biosynthesis of proline, since the ortholog of the ornithine aminotransferase (*OAT*, *Prupe_4G083300*) and a homolog of the pyrroline-5-carboxylate reductase (*PROC1*) were upregulated (**Table 1**). Proline level however remained stable, perhaps because of the simultaneous transcriptional activation of its degradation *via* Δ-1-pyrroline-5-carboxylate dehydrogenase (*Prupe_5G187700*, ortholog of *ALDH12A1*). The sulfur amino acid methionine and its precursor cystathionine were notably reduced after infestation (**Figure 3**, cluster 2.2.1. and **Supplementary Figure S6D**) and consistently, genes involved in methionine biosynthesis and degradation, *i.e.* the orthologs of *MS1* and *MGL*, were respectively down- and upregulated.

The branched-chain amino acids (BCAAs) isoleucine, leucine and valine were accumulated in Rubira tissues after infestation, as well as the isoleucine precursor threonine (**Figure 3**, cluster 1.1. and **Supplementary Figure S7D**). The genes coding for enzymes involved in early steps of biosynthesis were all downregulated by infestation: a homolog of *OMR1* and the ortholog of *ALS* (*Prupe_5G043100*) for isoleucine, orthologs of *IMDH2* and *DHAD* for leucine and valine respectively. On the contrary, homologs of *BCAT3*, encoding the enzyme catalyzing the last step of BCAAs biosynthesis, were upregulated (**Table 1**). Another discrepancy concerns lysine metabolism, since DEGs involved in its biosynthesis were downregulated while lysine and its catabolites, hydroxylysine and pipecolate, accumulated **Supplementary Figure S7E**. Beta-alanine and beta-aminoisobutyrate accumulation (BAIBA) (**Supplementary Figure S7F**) could be explained by the activation of the uracil degradation pathway (Parthasarathy et al., 2019), with the upregulation of the dihydropyrimidase homolog (*PYD2*) and Beta-ureidopropionase ortholog (*PYD3*, *Prupe_8G070200*).

Finally, it is worth mentioning the trace detection of aphid-induced alpha-aminobutyric acid (AABA) and sarcosine (N-methyl-glycine) (**Supplementary Figure S7F**). No DEG could be associated to these compounds.

#### Reconfigurations of secondary metabolism

Enrichment in the categories Phenylpropanoid metabolic process (GO: 0009698) and Flavonoid metabolic process (GO:0009812) underlines that significant modifications occurred in secondary metabolism in Rubira after infestation. The transcription of most DEGs implicated in the phenylpropanoid pathway was downregulated (**Table 1**). Upstream, the shikimic acid pathway genes involved in chorismic acid and aromatic amino acids were downregulated with the exception of the chorismate mutase 2 (*CM2*) ortholog (*Prupe_1G393400*). The two peach phenylalanine ammonia lyase (*PAL*) genes were not differentially expressed, but the *CYP73A5* ortholog, *Prupe_6G040400*, encoding a *trans*-cinnamate 4-monooxygenase (*C4H*) producing coumaric acid from cinnamic acid and an homolog of *4CL3*, encoding a coumarate-coA ligase specifically involved in flavonoid biosynthesis (Li et al., 2015), were both repressed. Homologs of genes involved in the biosynthesis of hydroxycinnamic derivatives, *HCT* (*Prupe_3G101400*), *C3’H* (*Prupe_1G580300*), and putative caffeoyl-CoA O-methyltransferases (*Prupe_2G199600*, *Prupe_2G199800*) were also downregulated, as well as downstream genes, operating at the entrance of the flavonoid pathway (ortholog of *CHS*, *Prupe_1G003000*) or for the subsequent methylation of flavonoids (*Prupe_2G199600* and *Prupe_2G199800*, homologs of *OMT1*, a multifunctional flavone 3’-O-methyltransferase also involved in formation of lignins and sinapoyl esters (Muzac et al., 2000). The only exception was the upregulation of the ortholog of *CAD4* (*Prupe_8G135300*) and a poorly annotated homolog of *CAD7*, coding respectively for cinnamyl alcohol dehydrogenases catalyzing the last step of monolignols biosynthesis (Tronchet et al., 2009) and for a NADPH-dependent aldehyde reductase converting (Z)-3-hexenal to (Z)-3-hexen-1-ol in Arabidopsis (Tanaka et al., 2018). The metabolites however present a different pattern: while most flavonoids did not change in response to infestation, many hydroxycinnamoylquinate derivatives were accumulated (**Figure 3**, cluster 1.1., **Supplementary Figure S8**; **Supplementary Table S10**). Aromatic amino acids increased, as well as the simple phenylpropanoids caffeic and isoferulic acids. Quinic acid esters increased while their direct precursor, free quinic acid, decreased. Many of the phenylpropanoids accumulated 48 hpi were caffeic conjugates, like caffeoyl-feruloyl-quinic acid and especially 5 isomers of dicaffeoylquinic acid. Finally, a few coumaroyl-acetyl-glucose esters increased, as well as the defense cyanogenic glycosides prunasin and caffeoyl-prunasin derived from phenylalanine (Shimomura et al., 1987; Yamaguchi et al., 2014). Interestingly, some of these compounds increased in Rubira whereas they decreased in the susceptible genotype GF305 after infestation (**Figure 3**, cluster 2.1. and **Supplementary Figure S4**).

## Discussion

In this work, we assessed the transcriptomic and metabolomic responses of peach to infestation by the green peach aphid GPA, a major global threat to horticultural and field crops. The study of peach is of particular interest since it is almost the only primary host of this polyphagous aphid species and peach accessions carry major resistance genes in contrast to secondary hosts of GPA. The responses of two rootstocks cultivars, GF305, susceptible to GPA and Rubira, carrying the major resistance gene *Rm2*, were studied 48 hpi, once the induced resistance of Rubira is fully established.

### GF305 and Rubira have highly contrasting responses to GPA infestation

Large differences in the number of DEGs induced by aphid infestation between susceptible and resistant genotypes are found in transcriptomic studies, but generally not to the same extent as in our investigation, which involved 5424 DEGs in Rubira, *i.e.* 20% of the predicted protein-coding sequences of peach genome, and only 35 DEGs in GF305. In peach, a previous study compared the transcriptomic response to GPA of the resistant peach cultivar “Fen Shouxing”, bearing the single dominant gene *Rm3*, to the response of a susceptible genotype (Niu et al., 2018). Infestation resulted in 1177 DEGs in the resistant line 48 hpi, compared to the aphid-free control plants, *i.e.* almost 5 times less than in the present study, and 282 in the susceptible line, that is 8 times more than in our work. Comparisons between studies should be made with caution since environmental conditions, plant age, genetic characteristics, initial number of aphids and RNAseq data filtering parameters, may not be identical across experiments. However, the much lower number of DEGs induced by infestation in the susceptible strain in our experiment could be explained by the very high susceptibility of GF305 to GPA (Sauge et al., 1998a), and/or by the high pre-adaptation of the aphids, since they were reared on this genotype before the experiment. Indeed, aphids continuously adapt their behavior to the plant characteristics, like their resistance level (Pompon and Pelletier, 2012; Jhou et al., 2021). Their experience on the plants they are reared on modulates aphids physiology temporarily, like duration of phloem intake, probing frequency, salivation duration (ten Broeke et al., 2014) and the gene expression level of particular effectors related to host utilization (Eyres et al., 2016; Thorpe et al., 2020). The second main difference concerns the number of repressed DEGs in the infested resistant line: Niu et al. (2018) found only a small proportion of repressed genes (a minimum of 6% 12 hpi and a maximum of 38% 72 hpi), whereas in our experiment half of the DEGs of the resistant genotype Rubira were repressed by infestation. This difference is due to the repression of hundreds of genes involved in metabolism and cell division (**Figure 5**) and is probably related to the strong inhibition of plant elongation measured post infestation in our experiment.

### GF305 weak response to infestation may reflect manipulation by GPA, repressing defense and promoting growth

The very limited transcriptional response of the susceptible genotype 48 hpi included only one underexpressed gene: *Prupe_5G025300*, a homolog of the CNL *RPM1*, a R-protein triggering HR-mediated defense against *P. syringae* (Kim et al., 2009). In Arabidopsis, RPM1 was found to interact functionally with the important defense protein RIN4, which is activated by an RPM1-induced protein kinase (RIPK) (Liu et al., 2009; Chung et al., 2011). *Prupe_5G025300* and 12 other *RPM1* homologs, as well as the *RIN4* and *RIPK* orthologs (*Prupe_8G19980* and *Prupe_7G198400* respectively), were upregulated in Rubira after infestation, which might lead to the detection of a particular aphid effector, thereby activating a set of defense responses controlled by *RPM1*. By contrast, *RPM1* downregulation and *RIN4* and *RIPK* not being differentially expressed in GF305 could traduce a neutralization of defense mechanisms by the aphid.

The induction of several growth-related hormones genes, specially brassinosteroid-related (e.g. *EXORDIUM*, *BEE3*, *KRP1*, (**Table 1** and **Supplementary Table S6**) in both susceptible and resistant genotypes are manifestations of the complex interplay between defense activation, resources allocation and growth that could either indicate a role in defense or the activation of a local physiological sink beneficial to the aphid (Züst and Agrawal, 2016). The metabolic profiles of GF305 did not show any significant change in the levels of primary metabolites that could improve the diet of the aphids, excepted maybe a decrease in glutamate (**Supplementary Table S9**), reported to lower the nutritional quality of phloem sap at high concentration (Karley et al., 2002). It is conversely possible that glutamate reduction impaired defense, since this compound is involved in long distance wounding signalling (Toyota et al., 2018). The observed decrease in the pool of defense compounds such as caffeoyl derivatives also supports the hypothesis of efficient manipulation of metabolism orchestrated by GPA.

Overall, the susceptibility was manifested by a quasi-absence of transcriptional response and a controlled inhibition of plant defenses by GPA in GF305, in contrast with the massive activation of defense responses observed in the resistant genotype.

### The Rm2 gene triggered PTI, ETI, SAR and HR markers upon infestation

**Figure 6:**
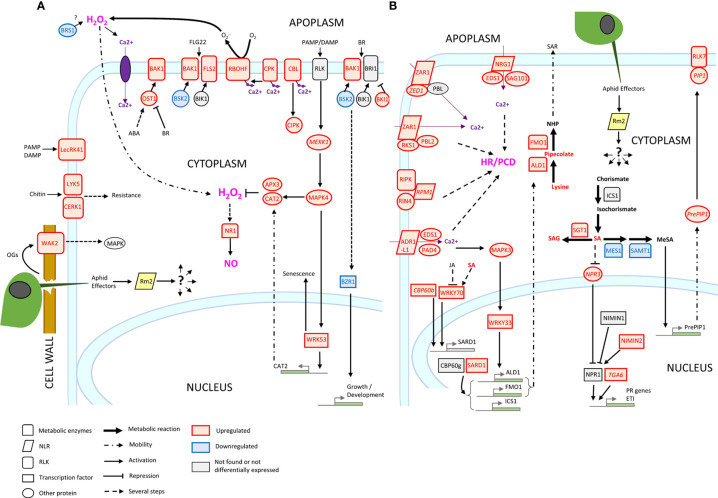
Transcriptional regulation of ETI, PTI, HR and SAR in Rubira 48 hours after infestation with GPA. **(A)** PTI genes. **(B)** ETI genes. Genes are annotated according to their Arabidopsis orthologs, non-orthologs (homologs) are noted in italics. Upregulated genes/metabolites are figured in red, downregulated genes/metabolites in blue and not found or not differentially expressed genes/metabolites in black. BIK1, Botrytis induced kinase 1; BRI1, Brassinosteroid insensitive 1; ICS1, Isochorismate synthase 1; NPR1, Nonexpresser of PR genes 1; OST1, Open stomata 1; RIN4, RPM1-interacting protein 4; RPM1-induced protein kinase; Disease resistance protein RPM1; PCD, programmed cell death; ADR1-LIKE 1, Activated disease resistance 1 like 1; ALD1, AGD2-like defense response protein 1; APX3, Ascorbate peroxidase 3; BAK1, Brassinoid insensitive 1-associated receptor kinase 1; BKI1, BRI1 kinase inhibitor 1; BRS1, BRI1 suppressor; BSK2, Brassinosteroid-signalling kinase 2; BZR1, Protein BRASSINAZOLE-RESISTANT 1; CAT2, Catalase 2; CBL10, Calcineurin B-like protein 10; CBP60B, Calmodulin-binding protein 60 B; CERK1, Chitin elicitor receptor kinase 1; CIPK25, CBL-interacting serine/threonine-protein kinase 25; CPK12, Calcium-dependent protein kinase 12; EDS1, Enhanced disease susceptibility 1; FLS2, Flagellin sensitive 2; FMO1, Probable flavin-containing monooxygenase 1; LECRK41, L-type lectin-domain containing receptor kinase IV.1; LYK5, LysM-containing receptor-like kinase 5; MEKK1, Mitogen-activated protein kinase kinase 1; MES1, Methyl esterase 1; MPK3, Mitogen-activated protein kinase 3; MPK4, Mitogen-activated protein kinase 4; NIMIN1, NIM1-interacting 1; NIMIN2, NIM1-interacting 2; NPR3, NPR1-like protein 3; NRG1, Probable disease resistance protein; PAD4, Lipase-like PAD4; PIP1, PAMP-induced secreted peptide 1; RBOHF, Respiratory burst oxidase homolog protein F; RLK7, Receptor like protein kinase 7; SAG101, Senescence associated gene 101; SAMT1, S-adenosylmethionine transporter 1; SARD1, Protein SAR deficient 1; SGT1, Salicylic acid glucosyltransferase 1; TGA6, TGACG motif-binding factor 6; WKRY33, Transcription factor WKRY33; WRKY53, Transcription factor WKRY53; ABA, Abscisic acid; SA, Salicylic acid; SAG, Salicylic acid glucoside; OGs, oligogalacturonides; PAMP, Pathogenesis-associated molecular pattern; DAMP, Damage-associated molecular pattern.

Three genes conferring high-level resistance to GPA, *Rm1*, *Rm2* and *Rm3*, have been detected in peach so far, located in the same genomic region at the bottom of chromosome 1 (Lambert and Pascal, 2011; Pascal et al., 2017; Niu et al., 2018). These three overlapping regions contain TNL candidate genes (Pan et al., 2022) which are likely receptors activated by recognition of a GPA saliva effector that triggers ETI. Our study suggests that *Rm2* activation by GPA effectors triggered coordinated processes in a dynamic network of PTI, ETI, pipecolate- and SA-mediated SAR contributing to aphid resistance through HR and defense metabolites accumulation (**Figure 6**).

Induction of peach homologs of cell surface receptors, potentiating elicitors detection such as flagellin (*FLS2*) chitin (*CERK1, LYK5*) and secreted peptides PIP1/PIP2 (*RLK7*), indicates that PTI was strongly activated. Among these induced RLKs, BAK1 is a key regulatory co-receptor required downstream of numerous immune responses through complex phosphorylation cascades of cofactors, whose implication in aphid detection has already been extensively demonstrated (Prince et al., 2014; Chaudhary et al., 2014; Vincent et al., 2017; Tungadi et al., 2021). BAK1 co-receptor interacts with other RLKs, like FLS2 and BRI1 and other co-receptors BKI1, BIK1 and BSKs, essential for BR-signalling transduction. We found an increase in the expression of *BKI1*, a negative regulator of *BRI1*, and a decrease of the positive regulator *BSK2* (Tang et al., 2011), suggesting a reduction of BZR1 dephosphorylation and thus of its activity as a positive regulator of BR-dependent gene expression (Ortiz-Morea et al., 2020). The downregulation of *BZR1* and *BSR1* indicates a general shutdown of BR pathways that could contribute to explain the slowdown of apices elongation measured 7 dpi. Furthermore, BRS1 was described as an apoplastic regulator of redox status and stress signalling (Zhang et al., 2021), while BAK1 also associates to OST1 to stimulate RBOHF and trigger ABA/NO regulated stomatal closure (Sierla et al., 2016). Several genes encoding wall-associated receptors, WAKs, were also induced. These proteins possess an extracellular domain that binds pectin (Kohorn et al., 2009) and are activated by oligogalacturonides (Brutus et al., 2010). Foyer et al. (2015), reported in a meta-analysis the induction of these receptors, in particular *WAK1* and *WAK2*, in tissues attacked by phloem-feeding insects. It has been proposed that aphid salivary effectors such as pectin-methylesterases and polygalacturonases released during stylet penetration between cells would produce oligogalacturonides, acting as DAMPS and detected by WAKs to trigger MAPK signalling cascades (Silva-Sanzana et al., 2020).

Downstream consequences of *Rm2* activation involved the helper RNLs ADR1-L1 and NRG1, whose peach orthologs were induced upon infestation of Rubira. ADR1 interacts with the lipase-like EDS1 and PAD4 to activate SAR and was recently found to localize at the plasma membrane by interacting with phospholipids, whereas NRG1 interacts with EDS1 and SAG101 to activate programmed cell death (Collier et al., 2011; Lapin et al., 2019; Saile et al., 2021). The peach orthologs of *EDS1, PAD4 and SAG101* induced in infested Rubira could thus play a major role in the activation of the resistance to GPA. This is supported by the induction of the ortholog of *SARD1* and of a close homolog of its regulator, *CBP60b*. The transcription factor SARD1 is thought to act downstream of EDS1 and PAD4 (Wang et al., 2011), by binding to the promoters of defense genes, notably *ALD1*, *FMO1* and *ICS1*, directly involved in the biosynthesis of pipecolate and SA (Sun et al., 2015). Consistently, Rubira showed a significant accumulation of SA and pipecolate 48 hpi, demonstrating the establishment of SAR: the biosynthesis of SA upregulates NHP biosynthesis genes, then the mobile NHP triggers *de novo* SA biosynthesis in systemic tissues and amplifies ETI and PTI (Schnake et al., 2020; Yildiz et al., 2021). A response similar to the one we report here has been already observed by Donze-Reiner et al. (2017) in switchgrass (*Panicum virgatum*) leaves infested by the greenbug *Schizaphis graminum*, suggesting that pipecolate could be a major defense player against aphids in very distant species. Moreover, the transcriptional activation of MPK3 and WRKY33 indicates that the SAR positive regulatory loop revealed by Wang et al. (2018a) and involving MPK3, WRKY33, ALD1 and pipecolate was also triggered in Rubira 48 hpi. The upregulation of several DEGs involved in SA catabolism (*SGT1*, *MES1*, *SAMT1*) or in the repression of its biosynthesis (*NPR3*), suggest a downregulation of SA, possibly as a feedback loop after a strong stimulation of its biosynthesis. Infestation also induced the expression of the different elements of the resistosome, including another NLR, coding for ZAR1, which does not require a helper (Adachi et al., 2019), but whose molecular association with kinases was recently discovered (Bi and Zhou, 2021): ZED1, a decoy pseudokinase that captures a *Pseudomonas* effector (Lewis et al., 2013) and ZAR1/RKS1, which has been shown to recognize PBL2 (Wang et al., 2015) and form a pentameric calcium-permeable complex associated to the membrane, the resistosome, that promotes cell death when activated by an effector (Wang et al., 2019).

Overall, our observations fit well with the emerging model of a strong interplay between ETI and PTI. Ngou et al. (2021) showed that a strong immune response requires both ETI and PTI, *via* upregulation of PTI signalling components at transcriptional and post transcriptional levels, ETI being insufficient to activate ROS production alone, but enhancing it upon elicitation by PAMPs and elevated protein levels of PTI signalling components. The same finding was made by Yuan et al. (2021), who showed that ETI could not be established without the activation of PTI, in particular the establishment of an efficient oxidative burst, with the PRRs and NLRs receptors working in synergy. Consistently, downstream signalling networks were activated in Rubira upon infestation, involving calcium-dependent proteins CBLs, CPKs, CIPK and NADPH oxidase, which can be inferred to have led to a strong production of apoplastic ROS, mitigated by the accumulation of detoxification enzymes. CPK2 is involved in ROS signalling through its direct interaction with RBOHD (Wang et al., 2018b) and CPK8, CPK9 and CPK12 participate, directly or indirectly, in H_2_O_2_ homeostasis (Zhao et al., 2011; Zou et al., 2015; Chen et al., 2019). The role of ROS in plant-aphid interaction is well documented (see Goggin and Fischer, 2022, for review). Interestingly, we found the specific activation of a RBOHF ortholog consistent with the work on Arabidopsis/GPA reported by Jaouannet et al. (2015), but different from Kuśnierczyk et al. (2007), who reported a transcriptional upregulation of RBOHD in Arabidopsis Cvi ecotype but not in Ws after infestation by GPA or *Brevicoryne brassicae*. There thus seems to be a specialization of the RBOHs in the defense against aphids, which would be dependent on plant genotypes. More implication of ROS in response to GPA was revealed by the upregulation of *Prupe_5G117000*, the ortholog of *WRKY53*. This transcription factor, inducible by H_2_O_2_, is a key regulator of senescence and controls the expression of catalase genes (Miao et al., 2004). Although direct essays of ROS in tissues are lacking here to formally establish the onset of HR, it is attested by the upregulation of the NO pathway, with upregulation of *NR1*, specifically responsible for NO generation under the control of ABA and H_2_O_2_ (Bright et al., 2006). Early NO induction was previously reported in wheat responding to the Russian wheat aphid (*Diuraphis noxia*) and nitrate reductase transcriptional upregulation was observed in Arabidopsis upon infestation by GPA and *B. brassicae* (Kuśnierczyk et al., 2007).

### Aphid-induced mobilization of glyoxylate and P5C/proline metabolisms likely promotes ROS burst in Rubira

**Figure 7:**
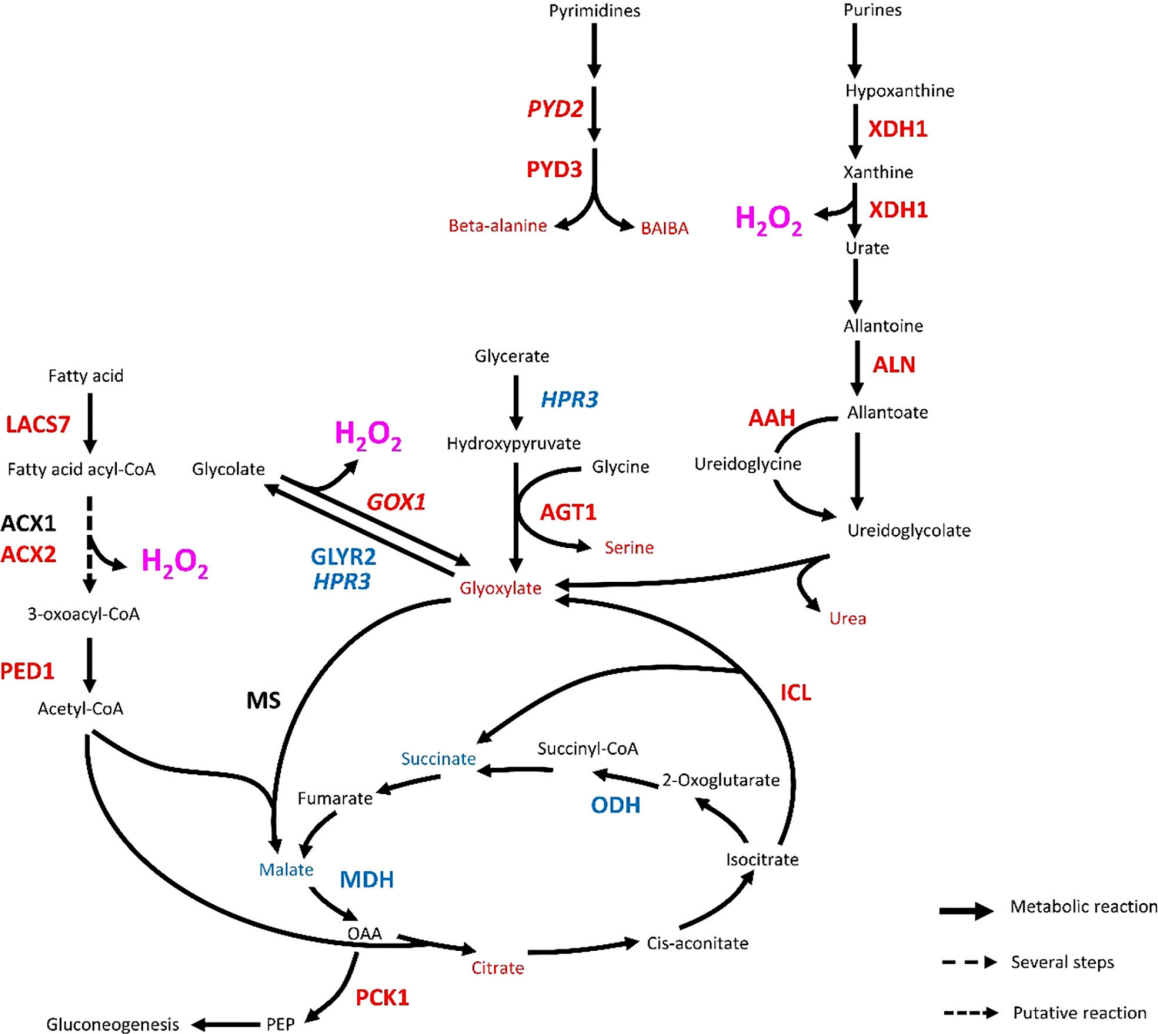
Transcriptional regulation of glyoxylate metabolic pathways in Rubira 48 hours after infestation with GPA. Genes are annotated according to their Arabidopsis orthologs, non-orthologs (homologs) are noted in italics. Upregulated genes/metabolites are figured in red, downregulated genes/metabolites in blue and not found or not differentially expressed genes/metabolites in black. AAH, Allantoate deiminase; ACX1, Peroxisomal acyl-coenzyme A oxidase 1; ACX2, Acyl-coenzyme A oxidase 2; AGT1, Glyoxylate aminotransferase 1; ALN, Allantoinase; GLYR2, Glyoxylate/succinic semialdehyde reductase 2; GOX1, Glycolate oxidase; HPR3, Glyoxylate/hydroxypyruvate reductase; ICL, Isocitrate lyase; LACS7, Long chain acyl-CoA synthetase 7; MDH, malate dehydrogenase; MS, Malate synthase; ODH, Oxoglutarate dehydrogenase; PCK1, Phosphoenolpyruvate carboxykinase 1; PED1, 3-ketoacyl-CoA thiolase 2, peroxisomal; PYD2, Dihydropyrimidinase; PYD3, Beta-ureidopropionase; XDH1, Xanthine dehydrogenase 1; BAIBA, Beta-aminoisobutyrate; PEP, Phosphoenol pyruvate.

An important metabolic consequence of aphid infestation in Rubira 48 hpi was the accumulation of glyoxylate and the upregulation of several afferent metabolic pathways (**Figure 7**). An activation of peroxisomal photorespiration was observed both at transcriptomic and metabolomic levels. Rojas et al. (2012) has shown the role of GOX2, which is a major source of H_2_O_2_, in the control of SA, JA and ethylene pathways, while Ahammed et al. (2018) demonstrated its role in resistance to *P. syringae* and its involvement in the regulation of SA-mediated signalling. Peroxisomal H_2_O_2_ production thus appears to be a key component of redox-mediated defense (Taler et al., 2004; Sandalio et al., 2021), and our data confirm the recent finding of its implication in Arabidopsis response to GPA (Xu et al., 2021). Our work suggests that the glyoxylate pool was also supplied by the glyoxylate shunt (**Figure 7**). Very little information is available about the involvement of this pathway in defense, but the upregulation of genes involved in lipid beta-oxidation feeding this pathway might be related to defense as well, since ACXs produce H_2_O_2_ (Kong et al., 2017) and ACX1 and PED1 are required for JA biosynthesis in the peroxisome (Cruz Castillo et al., 2004). Moreover, induction of *ICL* and *MS* have been reported in switchgrass infested by greenbugs (Donze-Reiner et al., 2017) and in soybean under attack by *B. cinerea* (Cots et al., 2002). It is therefore possible that the glyoxylate shunt operates in defense, beyond the fatty acid catabolism. Finally, the induction of purine and pyrimidine catabolism genes (homolog of *PYD2*; ortholog of *PYD3* and ortholog of *XDH1*) and the accumulation of uracil and thymine degradation products, i.e. beta-alanine and BAIBA (Zrenner et al., 2009), suggests the possibility that glyoxylate was also synthesized from urate and was a marker of an accelerated senescence (**Figure 7** and **Table 1**). Interestingly, the implication of *XDH1* in defense-related ROS generation has been reported in epidermal cells of Arabidopsis leaves under powdery mildew attack (Ma et al., 2016).

**Figure 8:**
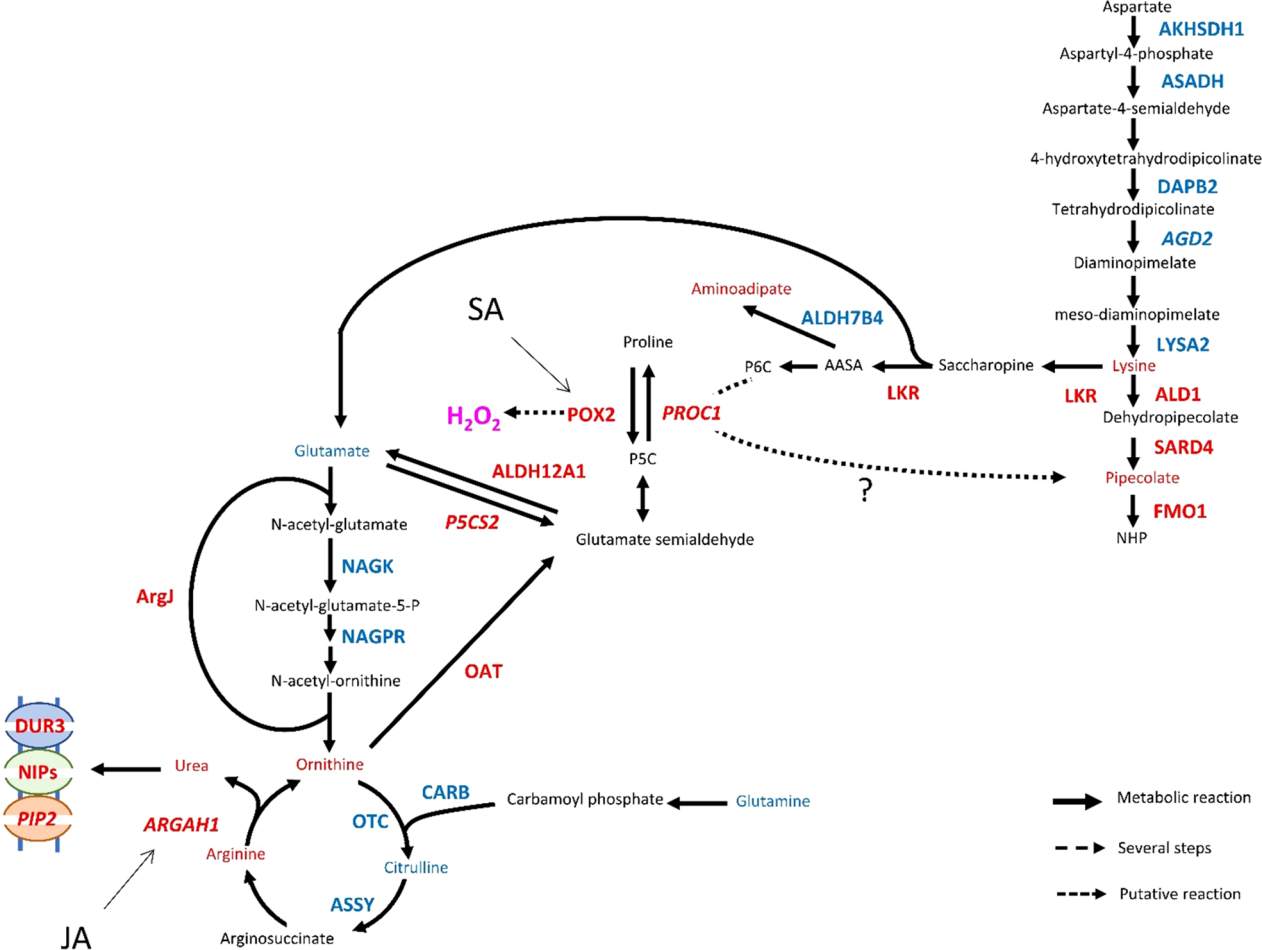
Transcriptional regulation of Arginine, proline and lysine metabolic pathways in Rubira 48 hours after infestation with GPA. Genes are annotated according to their Arabidopsis orthologs, non-orthologs (homologs) are noted in italics. Upregulated genes/metabolites are figured in red, downregulated genes/metabolites in blue and not found or not differentially expressed genes/metabolites in black. AGD2, LL-diaminopimelate aminotransferase; AKHSDH1, Aspartokinase/homoserine dehydrogenase 1; ALD1, AGD2-like defense response protein 1; ALDH12A1, Delta-1-pyrroline-5-carboxylate dehydrogenase 12A1; ALDH7B4, Aldehyde dehydrogenase family 7 member B4; ARGAH1, Arginase 1; ArgJ, Arginine biosynthesis bifunctional protein; Aspartate-semialdehyde dehydrogenase; ASSY, Arginosuccinate synthase; CARB, Carbamoyl-phosphate synthase large chain; DAPB2, 4-hydroxy-tetrahydrodipicolinate reductase 2; DUR3, Urea-proton symporter; FMO1, Probable flavin-containing monooxygenase 1; LKR/SDH, Alpha-aminoadipic semialdehyde synthase; LYSA2, Diaminopimelate decarboxylase 2; NAGK, Acetylglutamate kinase; NAGPR, N-acetyl-gamma-glutamyl-phosphate reductase; NIP1, Aquaporin Nodulin-26-like major intrinsic protein; OCT, Ornithine carbamoyltransferase; P5CS2, Delta-1-pyrroline-5-carboxylate synthase; PIP2, Aquaporin Plasma membrane intrinsic protein; POX2, Proline dehydrogenase 2; PROC1, Pyrroline-5-carboxylate reductase; SARD4, SAR deficient 4; AASA, alpha-amino adipate; NHP, N-hydroxypipecolate; P5C, Pyrroline-5-carboxylate; P6c, Pyrroline-6-carboxylate.

Other metabolic reconfigurations induced in Rubira by GPA infestation concern the arginine, proline and lysine pathways, which revolve around the Δ1-Pyrroline-5-Carboxylate (P5C). This compound is produced by the catabolism of proline, under the action of POXs, or by the conversion of glutamate into glutamic-γ-semialdehyde (GSA), the latter being in spontaneous equilibrium with P5C. A third biosynthetic pathway of P5C is the catabolism of ornithine through the action of OAT (Miller et al., 2009). All P5C biosynthetic pathways were transcriptionally activated 48 hpi, whereas *ALDH12A1* (*P5CDH*), which catalyzes its degradation to glutamate, was repressed (**Figure 8**). Furthermore, a net conversion of P5C to proline can be excluded as the proline pool remained stable: the simultaneous induction of *POX2* and *PROC1* (*P5CR*) rather indicate a high flux in the futile proline/P5C cycle. This cycle is thought to be ROS-generating because POX2 activity increases electron transfer from its cofactor FAD to the electron transfer chain and ultimately O_2_, resulting in mitochondrial ROS production (Miller et al., 2009). In animal cells, the proline/P5C cycle is central to the control of redox potential and cell death (Chalecka et al., 2021) and some studies have shown that it is involved in HR in plants (Monteoliva et al., 2014), in particular because the transcription of *POXs* in Arabidopsis was stimulated by SA and their activity was found increased in cells undergoing programmed cell death while the *pox* mutants, displaying reduced ROS levels and cell death, were more susceptible to *P. syringae* (Cecchini et al., 2011). These authors reported the same pattern as in our study: POX activation was accompanied by an increase in *P5CR* transcription, *i.e.* the proline/P5C cycle, but not *P5CDH*, while proline levels remained constant. The importance of OAT activity to PTI and ETI as well as to resistance to *P. syringae* has been demonstrated in *Nicotiana benthamiana* (Senthil-Kumar and Mysore, 2012). In our case, the urea cycle seemed to be oriented towards P5C formation, with induction of the ornithine-producing arginases *ARGAH1* and *ARGAH2*, and induction of *OAT*, generating P5C, while citrulline formation collapsed due to the repression of *OTC* and *CARB.* The urea cycle therefore appeared to operate in an open mode to convert arginine to P5C, which is consistent with increased susceptibility to clubroot disease (*Plasmodiophora brassicae*) and *B. cinerea* reported in the Arabidopsis *argah* mutants (Brauc et al., 2012; Gravot et al., 2012), and with the JA-dependant induction of arginase shown by Gravot et al. (2012). Another hypothetical role for PROC1 could be the biosynthesis of pipecolate. The ALD1 pathway is thought to be the only effective pipecolate pathway in plants but an alternative route exists, with 3 intermediates: saccharopine, α-aminoadipate-δ-semialdehyde (AASA), and Δ1 –piperidine-6-carboxylate (P6C) (Hartmann and Zeier, 2018). None of these compounds were detected in our study but a strong accumulation of α-aminoadipate was observed, which is a direct catabolite of AASA, and a gene coding for an enzyme of the saccharopine pathway was also upregulated: the lysine-ketoglutarate reductase/saccharopine dehydrogenase (*LKR/SDH*), that produces AASA. In human, P6C was found to be in spontaneous equilibrium with AASA and P5CR (PROC1/PYCR1) was proved to be able to turn P6C into pipecolate (Struys et al., 2014). Though not very likely, an involvement of this pathway in pipecolate accumulation after aphid infestation cannot be entirely ruled out. Finally, the accumulation of hydroxyproline (**Supplementary Figure S9**), which may be derived from turnover of parietal proteins, as well as pyrroline-3-hydroxy-5-carboxylate, suggests the existence of an infestation-stimulated hydroxyproline oxidase activity. Such an enzyme is known in animals (Cooper et al., 2008) but has no known equivalent in plants yet.

### Response to GPA activated protein recycling through autophagy in Rubira

The observed accumulation of lysine, arginine and ornithine contrasted singularly with the repression of genes involved in their biosynthesis (**Figure 8**). This was also true to some extent for BCAAs and histidine. The work of Niu et al. (2018) showed the similar repression of these biosynthetic pathways 48 hpi in the “Fen Shouxing” accession carrying the major GPA resistance gene *Rm3*. One might think that this repression followed a strong activation earlier, but the genes of the lysine biosynthetic pathway for example were all repressed between 6 and 72 hpi (Niu et al., 2018). The origin of these amino acids could therefore lie in the recycling of proteins from the infestation-induced reconfiguration of the proteome, or even from a state of senescence, as suggested by the accumulation of urea, which is frequently accumulated in this situation (Bohner et al., 2015). The upregulation of autophagy, revealed by the induced expression of *ATGs*, *NBR1* and genes involved in protein aggregate degradation (*CHIP* and *HSP70-4*) support the hypothesis of protein recycling. Interestingly, NBR1 was also found to target viral proteins, implying a possible coactivation of defenses against the aphid and the viruses it could transmit (Hafrén and Hofius, 2017).

### Metabolic depletion of key metabolites might have reduced the nutritional value of Rubira for GPA

Some amino acids pools diminished 48 hpi. This was the case for glutamine and methionine, in a manner consistent with the transcriptional regulation of their biosynthetic pathway. When the decrease in sorbitol is also considered, it appears that the aphid’s main sources of carbon, nitrogen and sulfur nutrition have been reduced in Rubira. Jordan et al. (2020) reported peach infestation levels by GPA proportional to their amino acid and carbohydrate content, stressing that their reduction has the potential to impair aphid growth, a similar conclusion to that of Karley et al. (2002), who compared aphid performance on young developing and old mature potato plants with contrasted nutritional value. The importance of plant nutritional quality was demonstrated in feeding choice tests of the specialists aphids *Uroleucon tanaceti* and *Macrosiphoniella tanacetaria* fed on tansy (*Tanacetum vulgare*), as both species showed their preference for plants highly fertilized with nitrogen, while infestation with *Uroleucon tanaceti* even increased phloem essential amino acids, especially methionine (Nowak and Komor, 2010; Jakobs et al., 2019). Infestation by sucking insects is well known to modify amino acid levels: GPA for example increases the amino acid/carbohydrate ratio in *Brassica pekinensis* sap, and the absolute amino acid content in whole leaves (Cao et al., 2016). This study even indicated that nutritional value was at least as important a criterion for food choice as the level of plant defense. It is therefore probable that the reduction in key nutrients and the coincidental slowing of plant elongation negatively impacted aphid performance on Rubira and contributed to limit colony settlement.

### Secondary metabolites accumulation may have contributed to antixenosis in Rubira

In compatible interactions, the very short exploration phase (only a few punctures before reaching the phloem), precedes a long ingestion phase as observed by Sauge et al. (1998b) on GF305 and a massive colonization associated with leaf curling. However, on Rubira, Weeping Flower Peach and “Fen Shouxing”, carrying *Rm2*, *Rm1*, *Rm3* resistance genes respectively, aphids perform numerous punctures before reaching the phloem in which they fail to feed for long periods (Sauge et al., 1998a; Niu et al., 2018). This leads eventually to the rejection of the plant as a host, a phenomenon characteristic of phloem-based antixenosis (Sauge et al., 1998a), described as well by Le Roux et al. (2010) in the interaction between GPA and various wild *Solanum* species. Antixenosis may result from the presence of soluble secondary metabolites and indeed we noted in Rubira a particular accumulation of caffeoyl derivatives of quinic acid. Many of them increased in Rubira while they decreased in GF305, albeit reaching equal levels due to their higher constitutive content of GF305. Other compounds were more abundant in Rubira, such as 3,5 dicaffeoylquinic acid, whose accumulation has already been demonstrated in infested Rubira (Poëssel et al., 2002). The deterrent effects of phenolic compounds on aphid feeding have been demonstrated *in vitro* (Dreyer and Jones, 1981). Antixenosis, measured by choice tests and manifested by the escape of aphids, or even antibiosis, manifested by a direct toxic effect of molecules on the insect, has also been demonstrated *in vivo*: in pepper (*Capsicum annuum*) the leaf contents of direct hydroxycinnamic acid derivatives, notably caffeic acid, increased after infestation by GPA (Florencio-Ortiz et al., 2021), while in peach a negative correlation was found between the content of phenolic compounds in leaves and the level of infestation by GPA (Jordan et al., 2020). Similarly, in apple, resistant cultivars showed higher levels of hydroxycinnamic acids, especially 4-caffeoylquinic acid (Berrueta et al., 2018). The mode of action of these compounds remains elusive. The presence of an orthodiphenol group on the caffeoyl residue makes them potential substrates for polyphenol oxidases and peroxidases in H_2_O_2_ detoxification reactions, as well as chelating agents for metals such as iron that can modulate the pro-oxidative Fenton reaction. Such metabolites could also feed the deposition of oxidized polyphenols along the stylet track, as shown by in the interaction between GPA and potato (Kerchev et al., 2012).

Concerning the phenolic pathways, there was an apparent contradiction between the transcriptomic and metabolomic data: we observed an overall repression of the phenolic pathway genes in Rubira 48 hpi, while aromatic amino acids clearly accumulated as well as some phenylpropanoids. This may result from the activation kinetics of the shikimate pathway, as Niu et al. (2018) showed a slight induction of this pathway genes in the first hours after infestation of “ Fen shouxing”, but then, 3 hpi, repression was gradually set up, until it reached a gene expression profile close to the one observed in Rubira 48 hpi. An alternative or complementary explanation is that the pathway was fed by aromatic amino acid originating from the activation of proteolysis/autophagy in infested tissues mentioned above.

Increased prunasin and its caffeoyl derivative may also have played a role in Rubira’s resistance, as suggested by the *in vitro* suppressive effect of prunasin on the duration of ingestion phase of bird cherry-oat aphid (*Rhopalosiphum padi*), whose primary host is the cyanogenic glycosides rich bird cherry (*Prunus padus*) (Halarewicz and Gabryś, 2012). On top of a direct toxic effect of cyanogenic glycosides, their turnover has also been shown to contribute to SA biosynthesis in peach (Diaz-Vivancos et al., 2017), thereby possibly reinforcing defense signalling.

## Conclusion

In conclusion, our results show that peach-GPA system provides a relevant model to study plant-aphid interactions and to decipher by omics approaches the molecular functions involved in susceptibility and in R-gene-mediated resistance.

**Figure 9:**
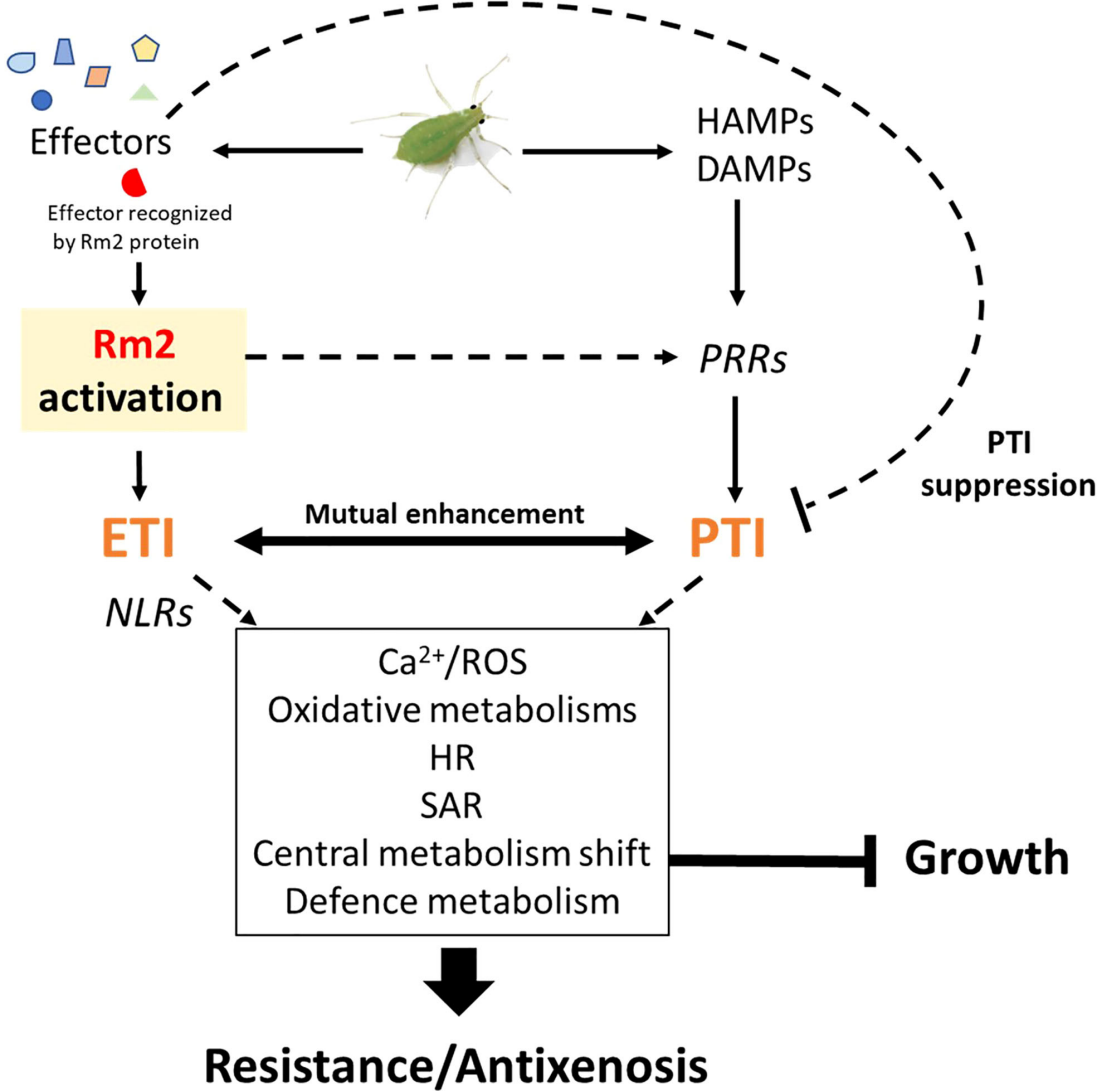
Conceptual model of the immune response to aphids in Rubira apices. Activation of the NLR receptor protein Rm2 by one of the *M. persicae* effectors triggers the transcription of numerous ETI and PTI genes as well as SAR and HR markers, resulting in metabolic reconfigurations favoring defense at the expense of growth: activation of H_2_O_2_-producing metabolic pathways, depletion of central metabolite pools and accumulation of caffeic acid conjugates.

These findings highlight the stealthy action of GPA on peach susceptible plants, likely resulting from repression of peach basal resistance by a very efficient effectors panel. They also illustrated the major transcriptome and metabolome reprogramming occurring during expression of the *Rm2*-mediated resistance, involving jointly ETI and PTI, SAR establishment by salicylic and pipecolic acids and primary and secondary metabolism changes resulting in enhanced defense (**Figure 9**). Further studies are needed to clarify which processes or defense compounds are key elements in triggering the antixenosis conferred by *Rm2* gene in peach.

## Data availability statement

The full dataset of transcripts is available in the NCBI Sequence Read Archive. (BioProject ID PRJNA877419): https://www.ncbi.nlm.nih.gov/bioproject/?term=PRJNA877419 Raw metabolomics dataset (accession number MSV000084377) can be downloaded from the publicly available MassIVE repository at the UCSD Center for Computational Mass Spectrometry website: https://massive.ucsd.edu/ProteoSAFe/dataset.jsp?task=56e8ba4bcb814e3d92863c88702f235c.

## Author contributions

RL, DR, J-LP designed the research and participated in setting up the experiments. PB performed analytical chemistry, datamining and contributed to write the paper. DR generated and analyzed the transcriptomic data. J-LP contributed to write the paper. RL, performed non-targeted metabolomics and wrote the paper. All authors contributed to the article and approved the submitted version.

## Acknowledgments

We warmly thanks Virginie Ledoux who took care of the Myzus persicae rearing and who contributed to experiments. This work took place in the experimental facilities of AHM INRAE Unit and we thank the staff for growing the plants.

## Conflict of interest

The authors declare that the research was conducted in the absence of any commercial or financial relationships that could be construed as a potential conflict of interest.

## Publisher’s note

All claims expressed in this article are solely those of the authors and do not necessarily represent those of their affiliated organizations, or those of the publisher, the editors and the reviewers. Any product that may be evaluated in this article, or claim that may be made by its manufacturer, is not guaranteed or endorsed by the publisher.

## Abbreviations

PAMPS, Pathogen-Associated Molecular Patterns; HAMPS, Herbivore-Associated Molecular Patterns; DAMPS, Damage-Associated Molecular Patterns; ETI, Effector Triggered Immunity, PTI, Pattern-triggered immunity; TNL, TIR Nucleotide-Binding and Leucine-rich repeat domains receptor; CNL Coiled-coil Nucleotide-Binding and Leucine-rich repeat domains receptor; DEG, Differentially expressed gene; hpi, hours post infestation; dpi, days post infestation; ROS, Reactive oxygene species; JA, Jasmonic acid; SA, Salicylic acid; ABA, Abscissic acid; BR, Brassinostéroïdes; NHP, N-hydroxypipecolate; GPA, Green peach aphid; HR, Hypersensible response; SAR, Systemic acquired resistance.
