# Supplementary material for: Molecular mechanisms of resistance to Myzus persicae conferred by the peach Rm2 gene: A multi-omics view
Source: Front Plant Sci. 2022 Oct 5;13:992544. doi: 10.3389/fpls.2022.992544 (PMC9581297; doi:10.3389/fpls.2022.992544)
Supplement: Supplementary file 1 [file Presentation_1.pptx]

## Slide 1
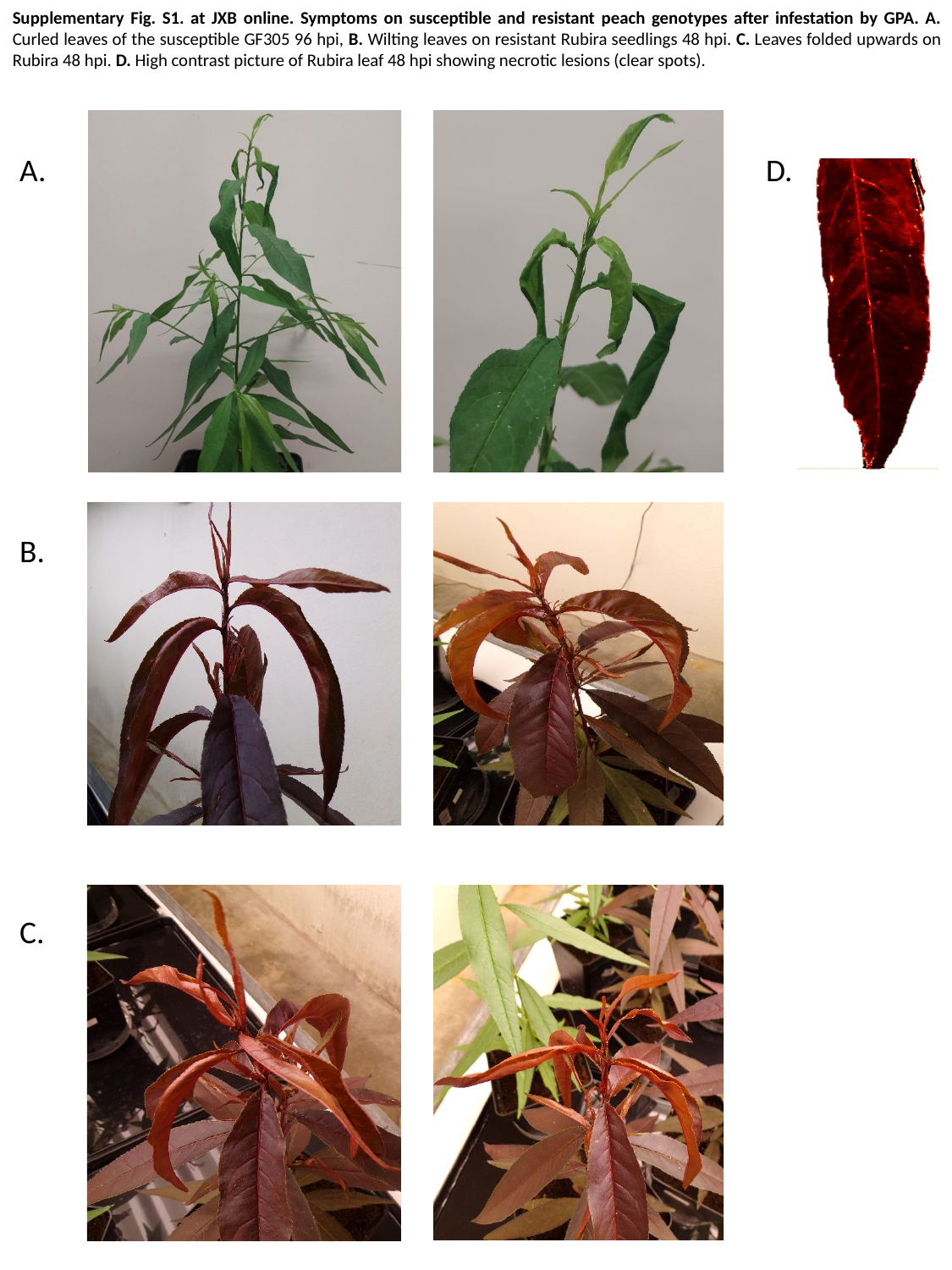

Supplementary Fig. S1. at JXB online. Symptoms on susceptible and resistant peach genotypes after infestation by GPA. A. Curled leaves of the susceptible GF305 96 hpi, B. Wilting leaves on resistant Rubira seedlings 48 hpi. C. Leaves folded upwards on Rubira 48 hpi. D. High contrast picture of Rubira leaf 48 hpi showing necrotic lesions (clear spots).
A.
B.
C.
D.

## Slide 2
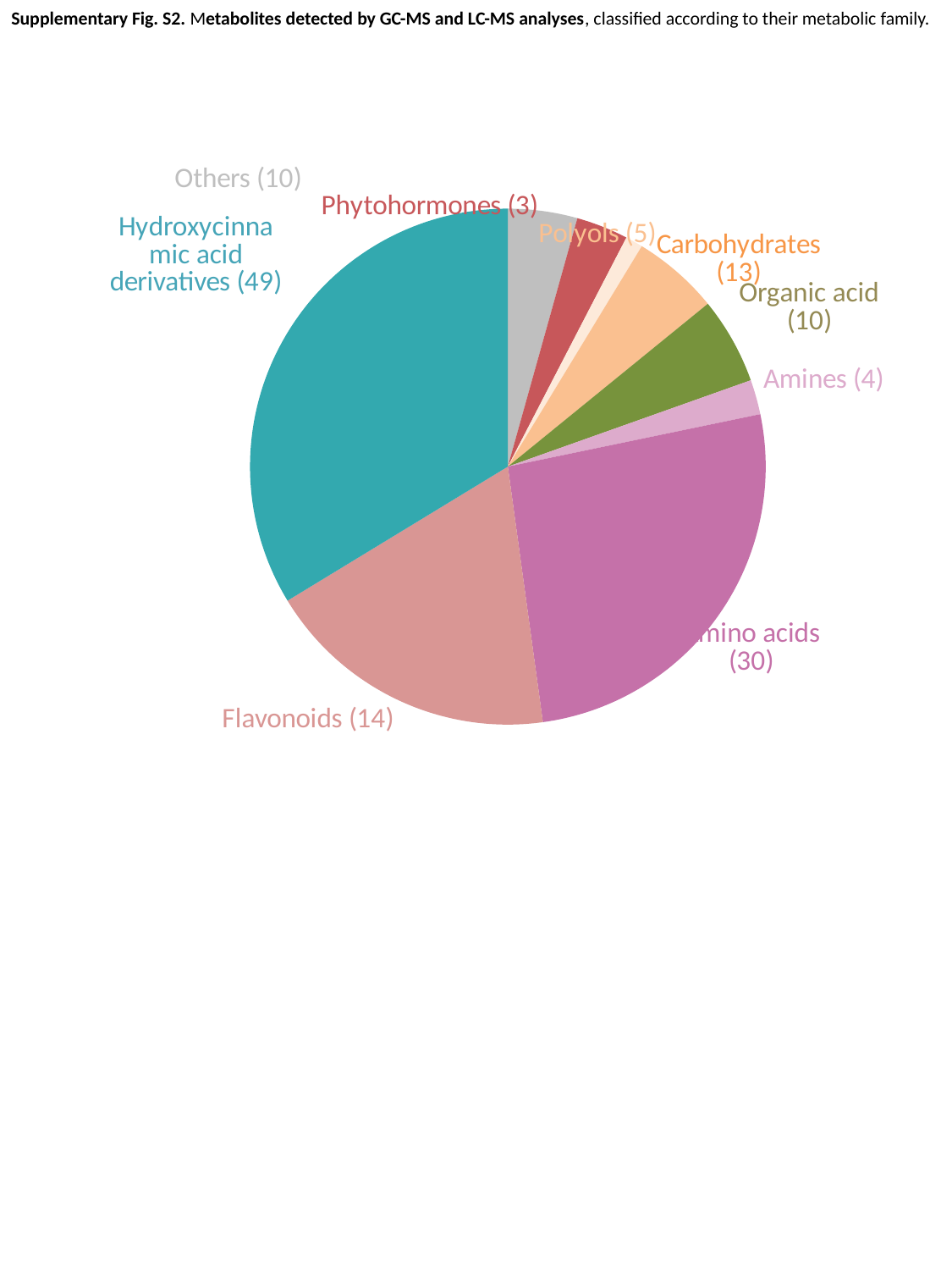

Supplementary Fig. S2. Metabolites detected by GC-MS and LC-MS analyses, classified according to their metabolic family.
### Chart
| Category | |
|---|---|
| Others | 4.0 |
| Phytohormones | 3.0 |
| Polyols | 1.0 |
| Carbohydrates | 5.0 |
| Organic acid | 5.0 |
| Amines | 2.0 |
| Amino acids | 24.0 |
| Flavonoids | 17.0 |
| Hydroxycinnamic acid derivatives | 31.0 |

## Slide 3
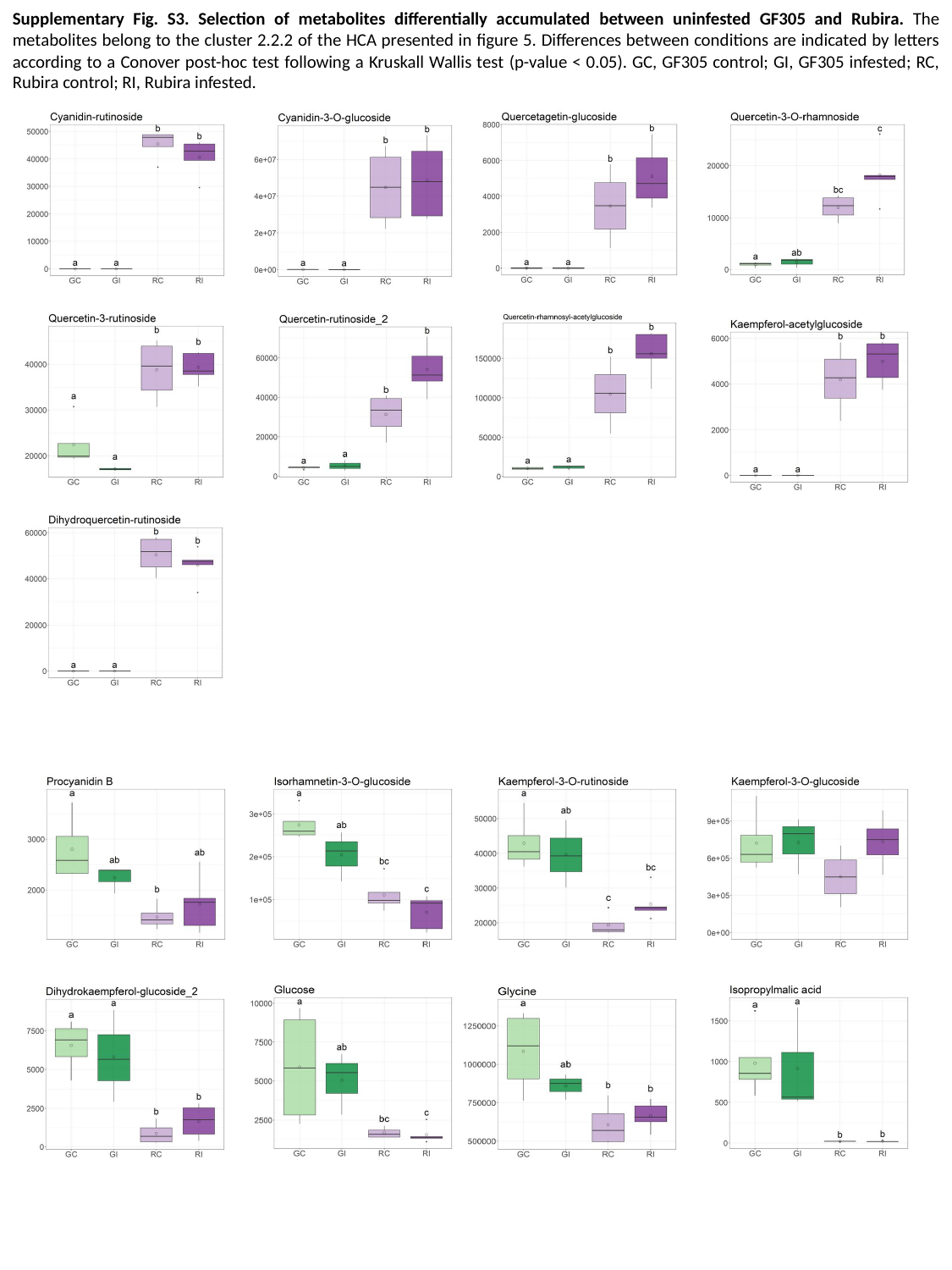

Supplementary Fig. S3. Selection of metabolites differentially accumulated between uninfested GF305 and Rubira. The metabolites belong to the cluster 2.2.2 of the HCA presented in figure 5. Differences between conditions are indicated by letters according to a Conover post-hoc test following a Kruskall Wallis test (p-value < 0.05). GC, GF305 control; GI, GF305 infested; RC, Rubira control; RI, Rubira infested.

## Slide 4
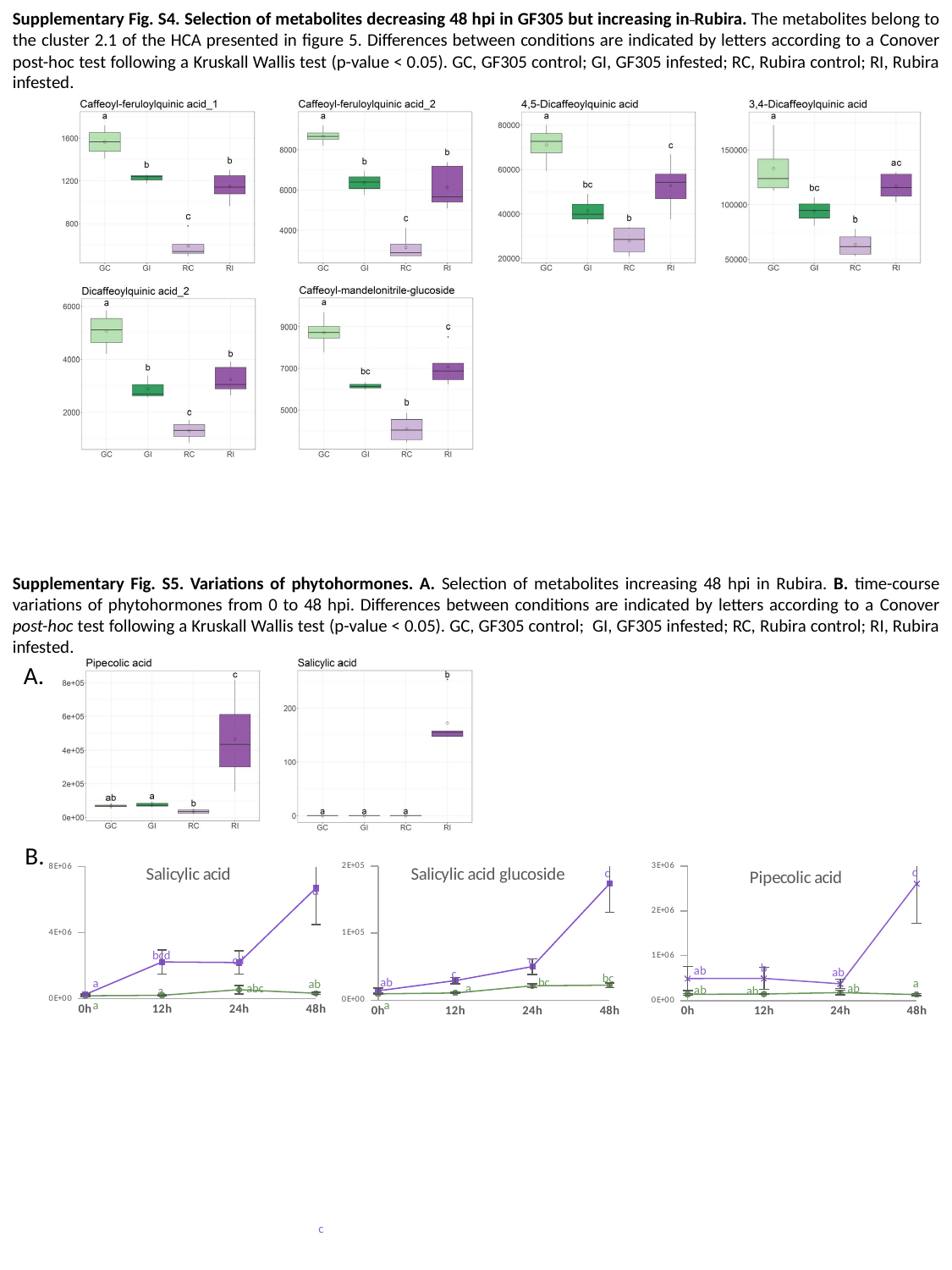

Supplementary Fig. S4. Selection of metabolites decreasing 48 hpi in GF305 but increasing in Rubira. The metabolites belong to the cluster 2.1 of the HCA presented in figure 5. Differences between conditions are indicated by letters according to a Conover post-hoc test following a Kruskall Wallis test (p-value < 0.05). GC, GF305 control; GI, GF305 infested; RC, Rubira control; RI, Rubira infested.
Supplementary Fig. S5. Variations of phytohormones. A. Selection of metabolites increasing 48 hpi in Rubira. B. time-course variations of phytohormones from 0 to 48 hpi. Differences between conditions are indicated by letters according to a Conover post-hoc test following a Kruskall Wallis test (p-value < 0.05). GC, GF305 control; GI, GF305 infested; RC, Rubira control; RI, Rubira infested.
A.
B.
### Chart: Salicylic acid
| Category | GF305 | Rubira |
|---|---|---|
| 0h | 179460.02925 | 255886.793 |
| 12h | 210901.9885 | 2229250.85 |
| 24h | 548248.5532 | 2194656.5061999997 |
| 48h | 330967.8906 | 6709453.5282 |d
bcd
cd
a
ab
abc
a
a
### Chart: Salicylic acid glucoside
| Category | GF305 | Rubira |
|---|---|---|
| 0h | 9369.435 | 13919.396 |
| 12h | 10641.29325 | 29159.565400000003 |
| 24h | 21440.8154 | 50204.7552 |
| 48h | 22370.4934 | 174100.6486 |c
c
c
bc
bc
ab
a
a
### Chart: Pipecolic acid
| Category | GF305 | Rubira |
|---|---|---|
| 0h | 137480.795 | 489711.99425000005 |
| 12h | 144880.6642 | 493368.18840000004 |
| 24h | 177034.73625 | 373169.99824999995 |
| 48h | 132061.64219999997 | 2608928.09375 |c
b
ab
ab
a
ab
ab
ab
c

## Slide 5
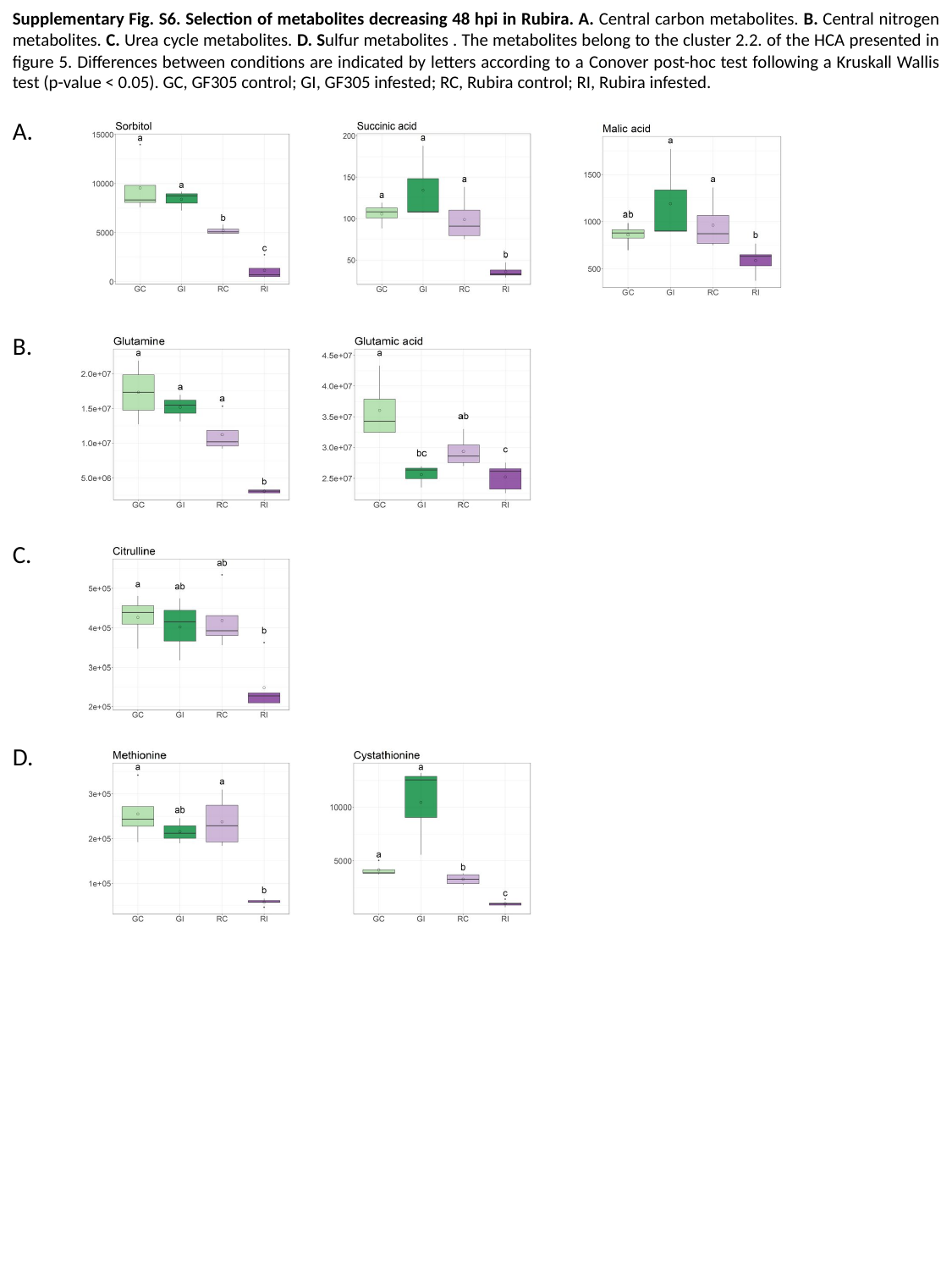

Supplementary Fig. S6. Selection of metabolites decreasing 48 hpi in Rubira. A. Central carbon metabolites. B. Central nitrogen metabolites. C. Urea cycle metabolites. D. Sulfur metabolites . The metabolites belong to the cluster 2.2. of the HCA presented in figure 5. Differences between conditions are indicated by letters according to a Conover post-hoc test following a Kruskall Wallis test (p-value < 0.05). GC, GF305 control; GI, GF305 infested; RC, Rubira control; RI, Rubira infested.
A.
B.
C.
D.

## Slide 6
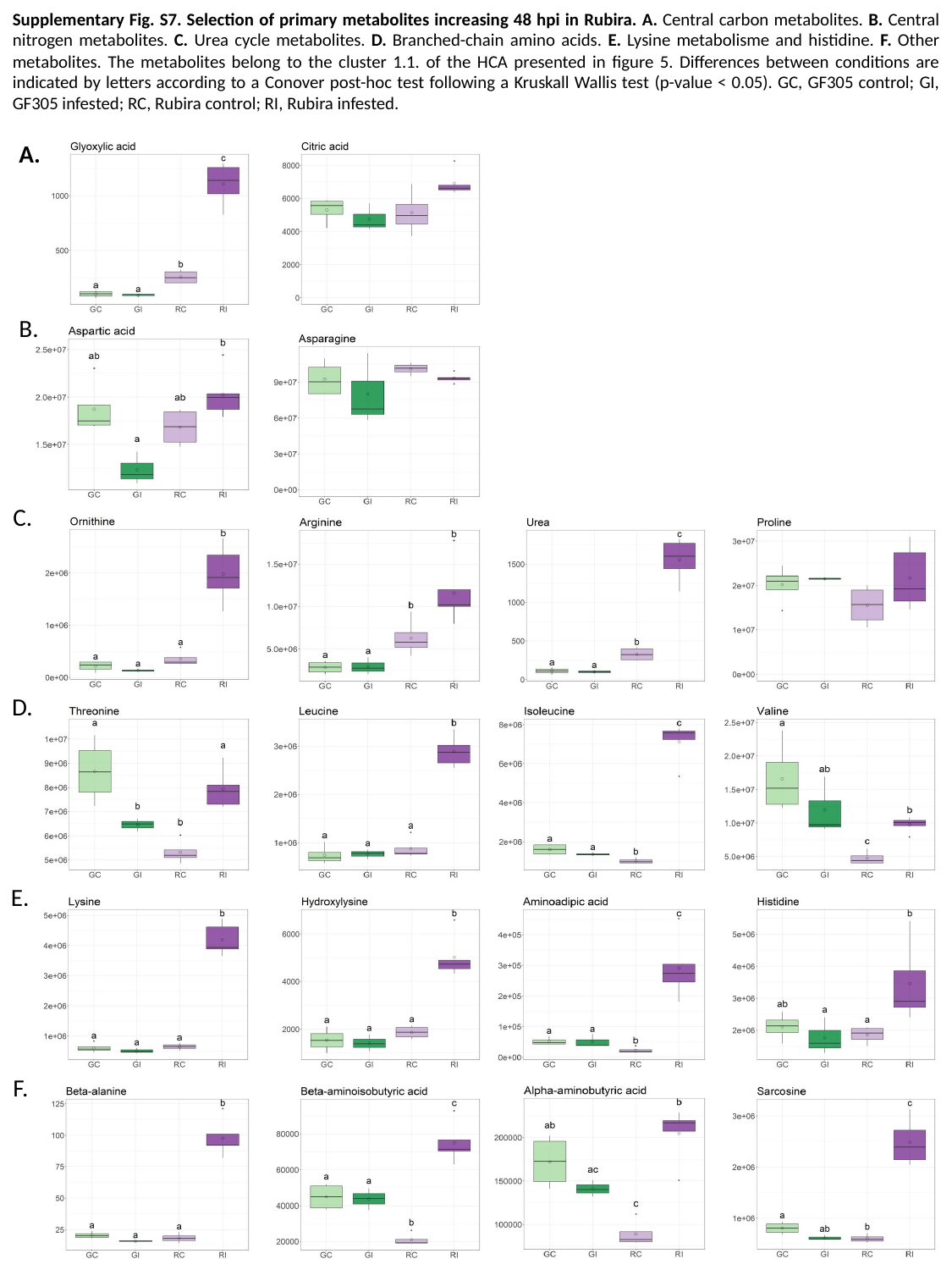

Supplementary Fig. S7. Selection of primary metabolites increasing 48 hpi in Rubira. A. Central carbon metabolites. B. Central nitrogen metabolites. C. Urea cycle metabolites. D. Branched-chain amino acids. E. Lysine metabolisme and histidine. F. Other metabolites. The metabolites belong to the cluster 1.1. of the HCA presented in figure 5. Differences between conditions are indicated by letters according to a Conover post-hoc test following a Kruskall Wallis test (p-value < 0.05). GC, GF305 control; GI, GF305 infested; RC, Rubira control; RI, Rubira infested.
A.
B.
C.
D.
E.
F.

## Slide 7
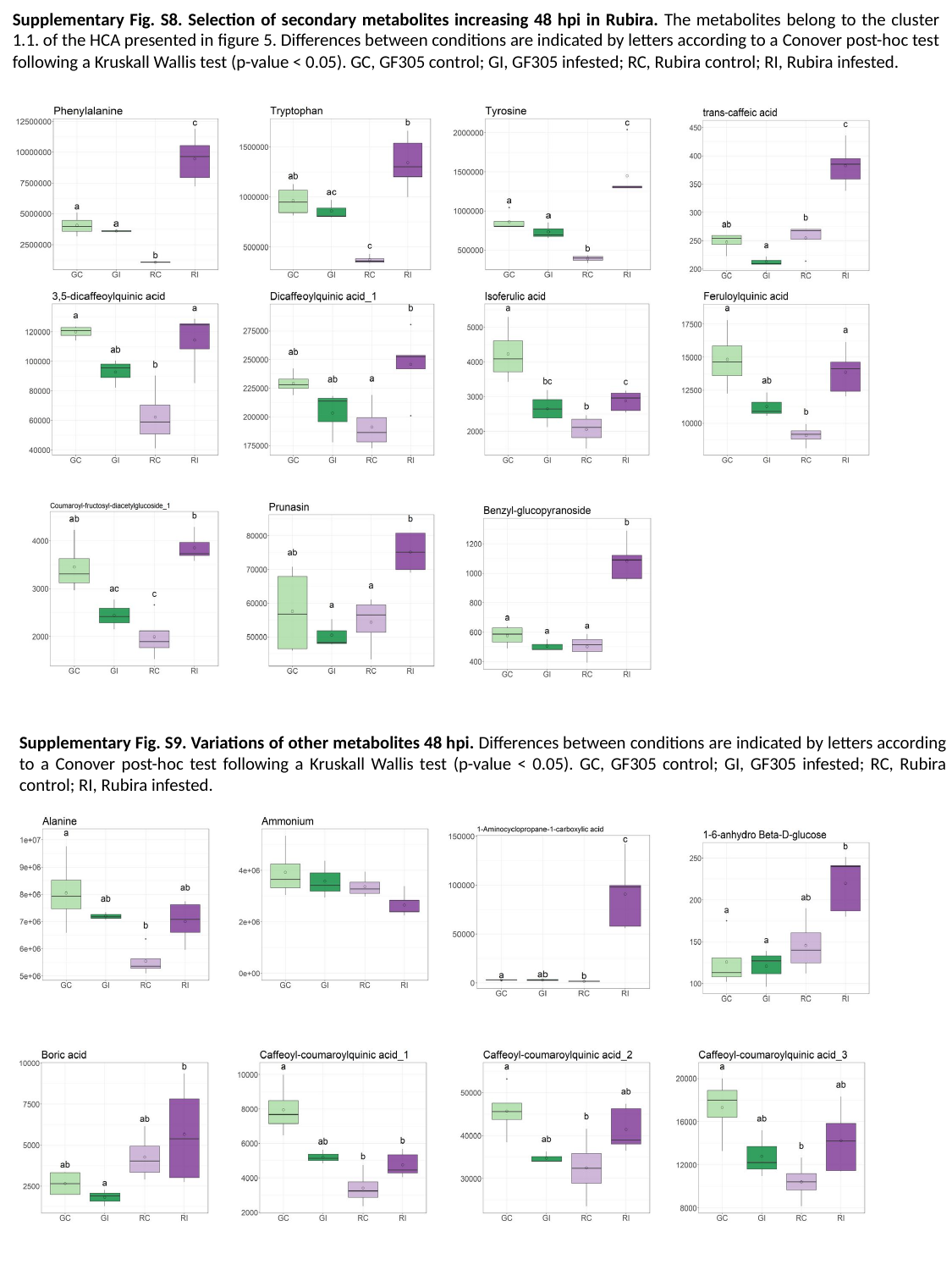

Supplementary Fig. S8. Selection of secondary metabolites increasing 48 hpi in Rubira. The metabolites belong to the cluster 1.1. of the HCA presented in figure 5. Differences between conditions are indicated by letters according to a Conover post-hoc test following a Kruskall Wallis test (p-value < 0.05). GC, GF305 control; GI, GF305 infested; RC, Rubira control; RI, Rubira infested.
Supplementary Fig. S9. Variations of other metabolites 48 hpi. Differences between conditions are indicated by letters according to a Conover post-hoc test following a Kruskall Wallis test (p-value < 0.05). GC, GF305 control; GI, GF305 infested; RC, Rubira control; RI, Rubira infested.

## Slide 8
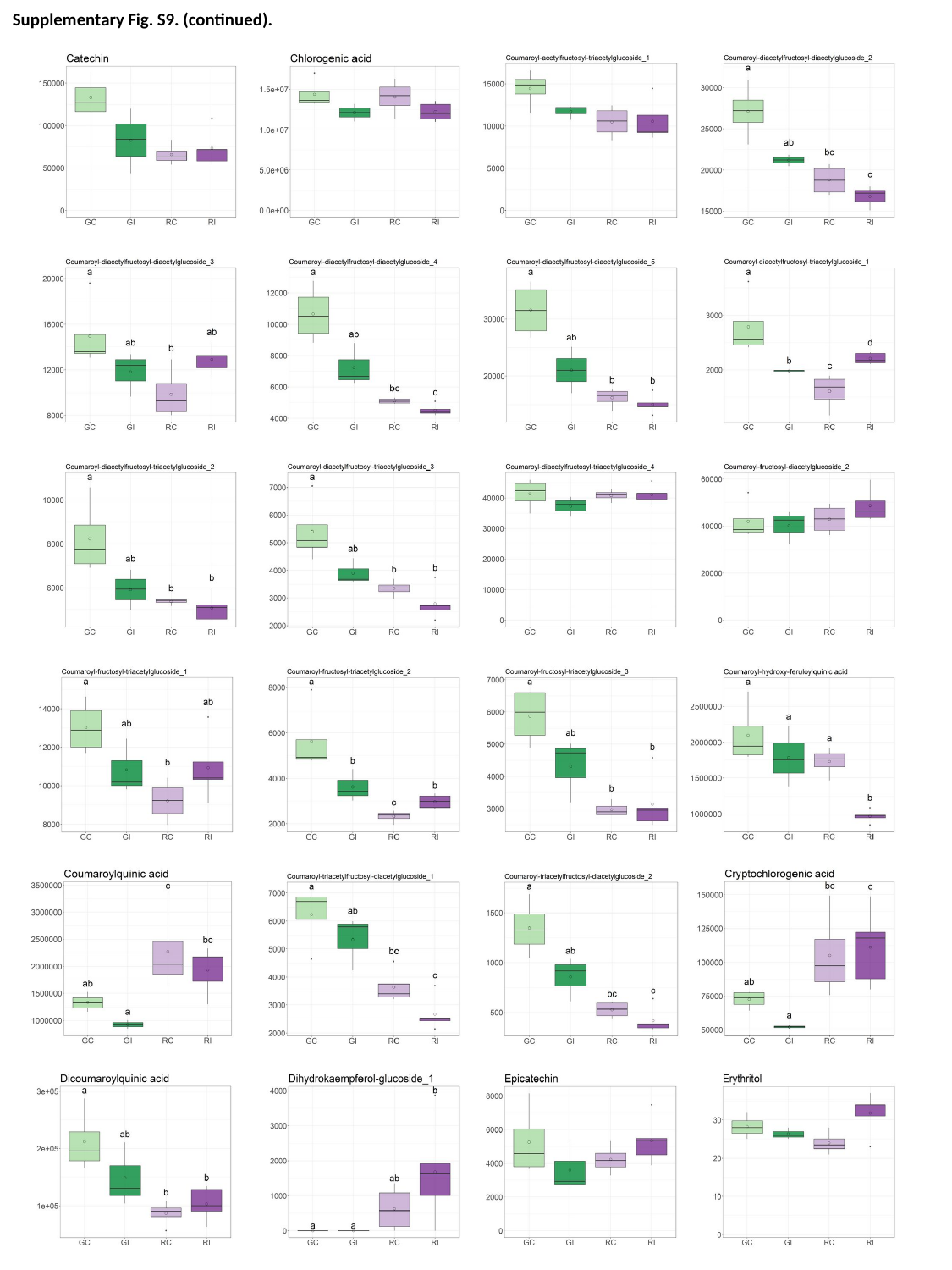

Supplementary Fig. S9. (continued).

## Slide 9
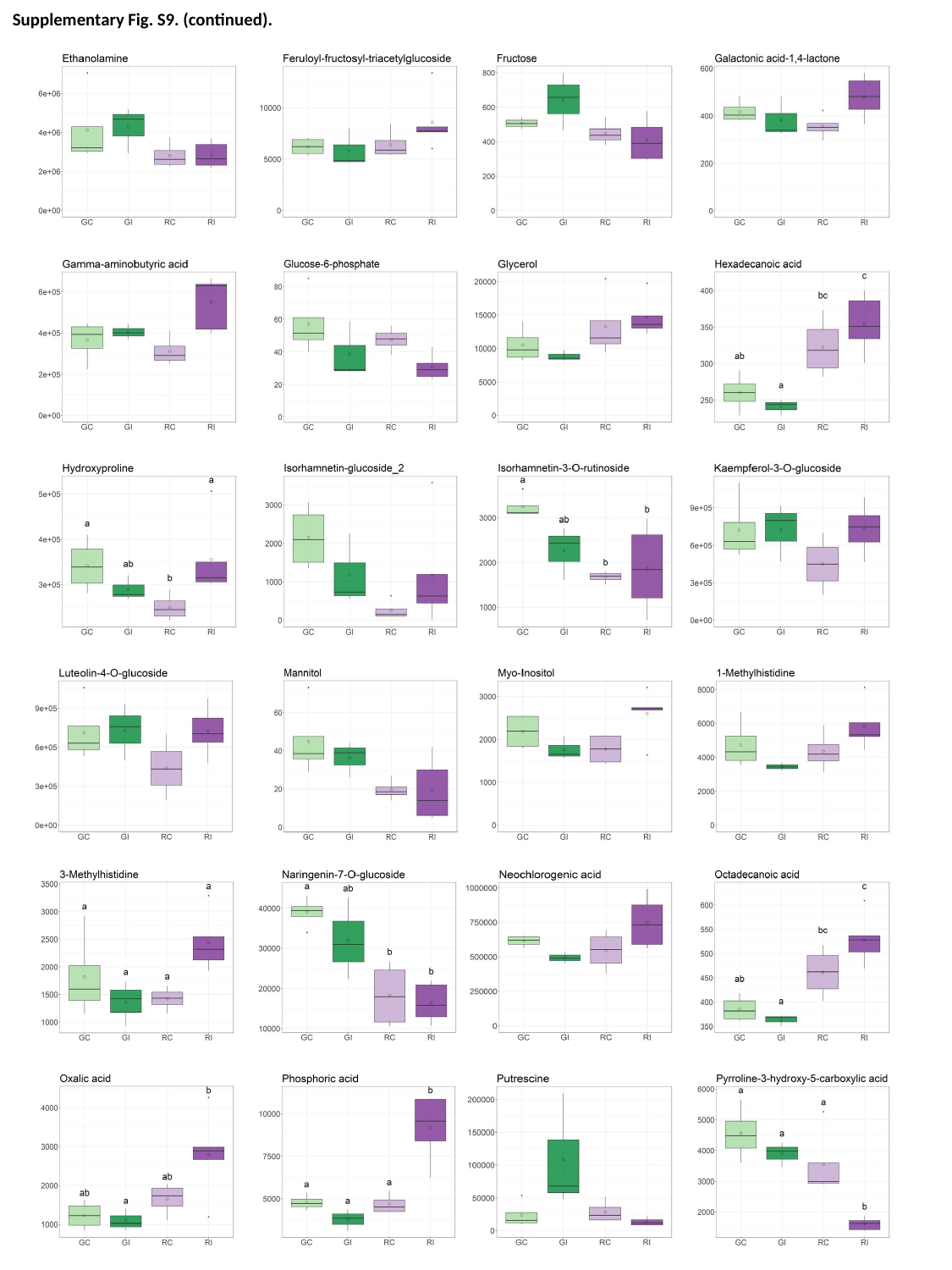

Supplementary Fig. S9. (continued).

## Slide 10
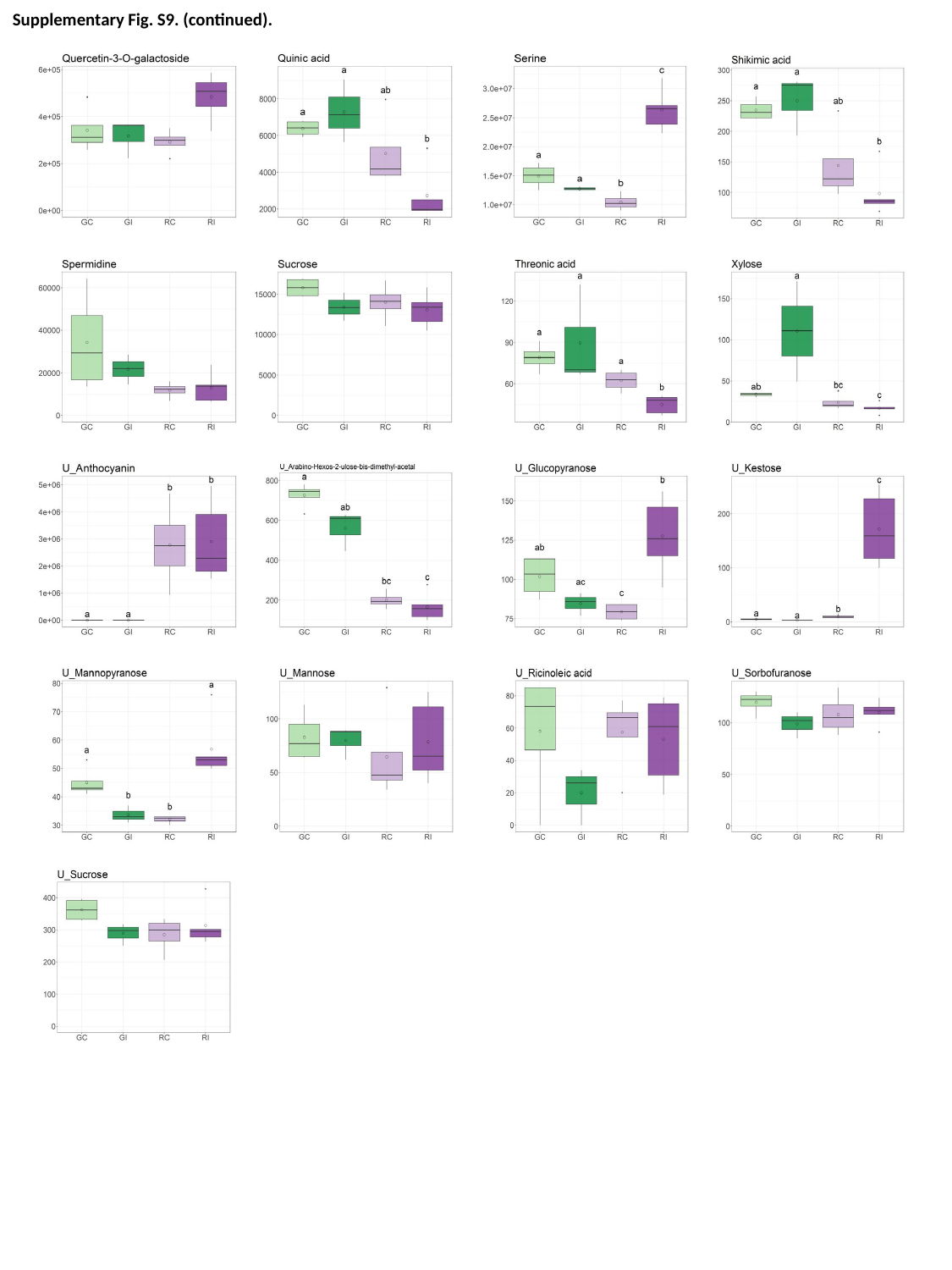

Supplementary Fig. S9. (continued).
